# Greater Thermal Plasticity Toward Heterogeneous Range‐Edge Environments of Three *Hypericum* Species

**DOI:** 10.1002/ece3.73486

**Published:** 2026-04-30

**Authors:** S. H. M. Koivusaari, M. H. Hällfors, J. Hjort, M.‐T. Hyvärinen, M. Levo, M. Luoto, C. Møller, Ø. H. Opedal, L. Pietikäinen, A. Romero‐Bravo, A. L. K. Mattila

**Affiliations:** ^1^ Department of Geosciences and Geography University of Helsinki Helsinki Finland; ^2^ Botany and Mycology Unit, Finnish Museum of Natural History University of Helsinki Helsinki Finland; ^3^ Finnish Environment Institute Helsinki Finland; ^4^ Research Centre for Ecological Change, Faculty of Biological and Environmental Sciences University of Helsinki Helsinki Finland; ^5^ Geography Research Unit University of Oulu Oulu Finland; ^6^ Organismal and Evolutionary Biology Research Programme, Faculty of Biological and Environmental Sciences University of Helsinki Helsinki Finland; ^7^ Department of Plant and Environmental Sciences University of Copenhagen Copenhagen Denmark; ^8^ Department of Biology Lund University Lund Sweden; ^9^ Department of Ecology and Evolution, School of Life Sciences University of Sussex Brighton UK

**Keywords:** climatic variability hypothesis, *Hypericum maculatum*, *Hypericum montanum*, *Hypericum perforatum*, phenology, species' distributions

## Abstract

Intraspecific variation in phenotypic plasticity can affect the ability of populations, and thus species, to respond to environmental changes. However, the prevalence and drivers of such variation are not well known. Most proposed explanations for intraspecific variation in phenotypic plasticity involve mechanisms associated with a population's position within the species' geographic range or the environmental heterogeneity experienced by the population. To assess the effect of these two drivers, and their potential interaction, we use a combination of germination and greenhouse experiments to measure thermal phenotypic plasticity in traits ranging from germination probability to flower abundance in populations of three *Hypericum* species sampled across their European ranges. We then relate thermal plasticity to each population's position within the species' range and to the environmental heterogeneity of the sampling site. Our results revealed that, while average thermal plasticity in several traits was similar among the three tested *Hypericum* species, it varied among the conspecific populations. Specifically, populations closer to the range edge tended to be more plastic in germination probability and plant height, while populations from more heterogeneous environments tended to be more plastic in flowering phenology, plant height, and flower abundance. Interestingly, for plasticity in germination phenology, plant height, and flower abundance, we found a substantial interactive effect with accentuated plasticity in heterogeneous sites near the range edge. This suggests that populations in heterogeneous environments at range edges may adjust to environmental change via phenotypic plasticity more effectively than do other conspecific populations. These results support both tested drivers and reveal important interactive patterns for some of the tested traits. Furthermore, they encourage further research on plasticity that considers both range position and environmental heterogeneity.

## Graphical abstract

Predictions from the highest‐ranked models testing the effect of range position (RP) and environmental heterogeneity (EH) on trait plasticity, with population‐specific plasticity values in blue (*Hypericum montanum*), red (*H. perforatum*), and green (*H. maculatum*). For RP and EH metrics, value −1 indicates the lowest distance to the edge/lowest amount of environmental heterogeneity and value 1 the greatest distance to edge/highest amount of environmental heterogeneity. A, B germination probability; C–E germination phenology; and F, G flowering phenology; H–J plant height and K–M flower abundance. Statistically significant *p*‐values are shown with *p* < 0.01 = **; *p* < 0.05 = *; *p* < 0.1 = +.

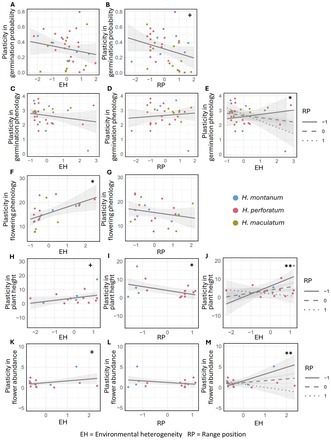

## 1 Introduction

Phenotypic plasticity is the genotype's ability to produce different phenotypes in different environments (Pigliucci 2001). In the context of global change, phenotypic plasticity is a crucial mechanism by which organisms can quickly adjust to variable and temporally unfavorable environmental conditions. Such adjustment can give populations time to disperse to more favorable areas or to adapt to changing conditions in their current habitats, thereby increasing long‐term survival (Jump and Peñuelas 2005; Nicotra et al. 2010). The degree of phenotypic plasticity can vary not only among species but also among conspecific populations (Matesanz and Ramírez‐Valiente 2019; Van Tienderen 1991). Furthermore, the amount of intraspecific variation can vary among species and traits (Hällfors et al. 2025; Schoen and Brown 1991). Such intraspecific variation in phenotypic plasticity can affect the ability of a species to respond to environmental changes, such as climate change (Valladares et al. 2014). While there is an increasing effort to quantify intraspecific variation in phenotypic plasticity, such data remain scarce, largely because obtaining the necessary data often requires resource‐intensive experiments (de la Mata and Zas 2023; Fox et al. 1999; Matesanz and Ramírez‐Valiente 2019; Molina‐Montenegro and Naya 2012; Møller et al. 2025; Oostra et al. 2018). Collecting data from a wide range of species and traits to assess intraspecific patterns in phenotypic plasticity will help address this gap and allow more reliable forecasts of biotic resilience in the event of environmental changes (Opedal et al. 2015; Valladares et al. 2014).

Given the scarcity of data, the drivers of intraspecific variation in phenotypic plasticity remain poorly understood. Studies aiming to explain intraspecific patterns in phenotypic plasticity have often assumed that phenotypic plasticity varies within a species' range (e.g., Mägi et al. 2011; Zettlemoyer and Peterson 2021). More specifically, plasticity has been proposed to differ between populations located at the core versus at the periphery of a species range, with either core populations being more plastic than peripheral populations (Mägi et al. 2011), or *vice versa* (Zettlemoyer and Peterson 2021). Several hypotheses have been proposed for why such patterns would emerge (reviewed by Usui et al. 2023). Some of these hypotheses relate to expected abundance patterns across species' ranges. The abundance of a species is traditionally assumed to be highest at the core of a species range and decline toward the periphery, due to declining suitability of environmental conditions toward the periphery (Brown 1984). As a result of more individuals and greater gene flow, core populations may possess more genetic variation allowing for greater phenotypic plasticity to evolve (Arnaud‐Haond et al. 2006; Ellstrand and Elam 1993). On the other hand, gene flow from core populations could also dilute local adaptation and reduce fitness in peripheral populations (i.e., cause gene swamping) (Usui et al. 2023). This can, rather counterintuitively, result in greater plasticity in peripheral populations if the evolution of plasticity is enhanced to cover fitness losses (Chevin and Lande 2011).

Another hypothesis for how and why plasticity would vary between core and peripheral populations relates to differences in environmental heterogeneity (Zettlemoyer and Peterson 2021). Whether occurring in space or in time, environmental heterogeneity is often expected to lead to selection for greater phenotypic plasticity (Alpert and Simms 2002; Baythavong 2011; Gianoli 2004; Gianoli and González‐Teuber 2005; Sultan and Spencer 2002; Zettlemoyer and Peterson 2021, but see e.g., Dupont et al. 2025; Leung et al. 2020; Siljestam and Östman 2017). Several theoretical studies have found support for this expectation (Eriksson and Rafajlović 2022; Scheiner 2013; Sultan and Spencer 2002), but empirical studies where the position of a population within the species' range (hereafter, range position) has been used as a proxy for the amount of environmental heterogeneity experienced by the populations, have yielded inconsistent results (Dobson and Zarnetske 2025; Gunderson and Stillman 2015; Kotilainen et al. 2024; Mitchell et al. 2011; Molina‐Montenegro and Naya 2012). The use of a population's range position as a proxy for environmental heterogeneity and thus phenotypic plasticity has often been motivated by the Climatic Variability Hypothesis (CVH, Addo‐Bediako et al. 2000). According to this hypothesis, climatic heterogeneity, and thus phenotypic plasticity, should increase toward the poleward edges of a species' range. Due to the lack of evidence for such a latitudinal pattern in phenotypic plasticity, however, recent studies have suggested that phenotypic plasticity could be explained by variation in environmental heterogeneity occurring at a smaller scale (Manenti et al. 2017; Noer et al. 2022). Such variation may disrupt, or even override, latitudinal patterns in climatic conditions (Potter et al. 2013). Accounting for local‐scale indicators of environmental heterogeneity may thus be critical to accurately predict patterns in plasticity (Potter et al. 2013; Usui et al. 2023).

Here, we quantify thermal phenotypic plasticity across a set of European populations of three *Hypericum* species. We compare species' average responses to temperature, intraspecific variation in those responses, and how the amount of intraspecific variation differs among species. Then, we ask whether and how (1) populations' range position, and (2) the amount of environmental heterogeneity at the source locations of the populations are related to the degree of thermal plasticity, and (3) if there are additive or interactive effects of these two factors on plasticity. By jointly evaluating the impact of range position, environmental heterogeneity, and their interaction, we aim to avoid the common pitfalls related to only testing a single expectation or using range position as a proxy for environmental heterogeneity. Furthermore, this approach allows us to test how local‐scale spatial environmental heterogeneity affects phenotypic plasticity and to uncover potential interactive effects on variation in plasticity, both of which have rarely been empirically tested (but see Baythavong 2011; de la Mata et al. 2022 for a case of testing the independent impact of spatial environmental heterogeneity on plasticity). We expect to find greater plasticity either in core or peripheral populations, and in more heterogeneous environments. Furthermore, based on possible alternative influences of genetic diversity and gene flow in the peripheral populations, we expect that the positive effect of increased environmental heterogeneity depends on the populations' range position either by dampening or enhancing the effect in peripheral populations.

## 2 Methods

### 2.1 Study Species and Populations

*Hypericum montanum* (L.), *H. perforatum* (L.) and *H. maculatum* (Crantz) are perennial herbs of the Hypericaceae family. The native ranges of *H. perforatum* and *H. maculatum* cover most of Europe (Hultén and Fries 1986) and they are commonly found in grassland habitats (Hypericum perforatum L. in GBIF Secretariat 2023; Hypericum maculatum Crantz in GBIF Secretariat 2023). *Hypericum montanum*, on the other hand, has a more restricted distribution (Hultén and Fries 1986) and occurs mainly in woodland habitats (Hypericum montanum L. in GBIF Secretariat 2023). While *H. perforatum* is facultatively apomictic, *H. montanum* and *H. maculatum* are obligate sexual reproducers (Matzk et al. 2003). All three species have relatively limited dispersal capacity (dispersal distance of 1–5 m for 50% and 99% of the seeds; Lososová et al. 2023).

**FIGURE 1:**
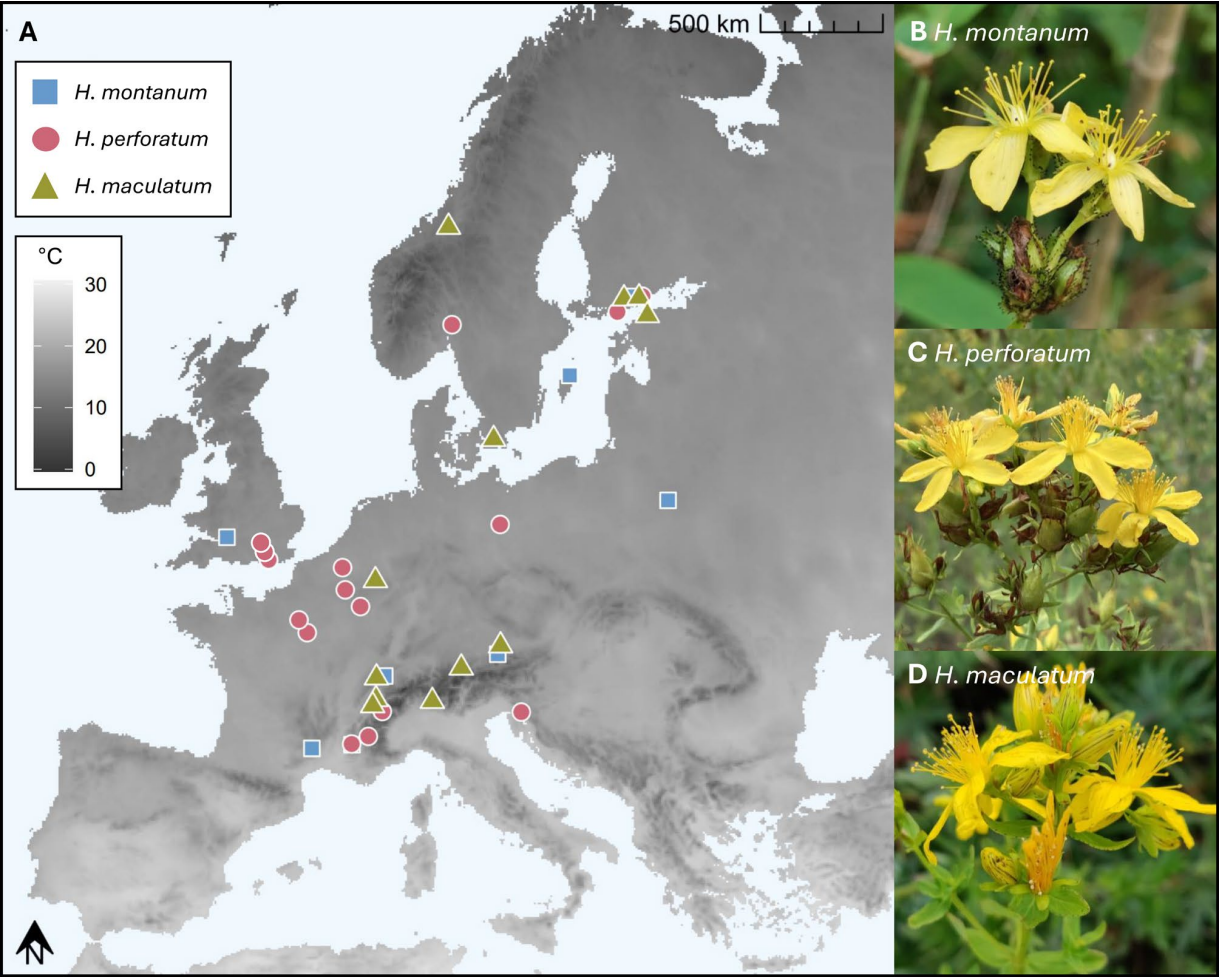
Locations of the seed collection sites of the three studied *Hypericum* species in Europe (A) and pictures of the study species (B‐D). See Table S1 for more details on the seed collection sites. The mean temperature of the warmest quarter (°C) (WorldClim; Fick and Hijmans 2017) is shown in the background of A. Photo credit of *H. montanum* : https://www.inaturalist.org/observations/88095325, *H. perforatum* : https://www.inaturalist.org/observations/172389923, *H. maculatum* : https://inaturalist.ca/observations/53887149.

We acquired seeds of the three species from different parts of their native ranges, totaling 8 *H. montanum* , 18 *H. perforatum* , and 12 *H. maculatum* populations (after filtering as described in Section 2.5), either by collecting them in the field or acquiring seed accessions from European seed banks (Figure 1, Table S1). The seeds were primarily collected between 2017 and 2021, with one *H. perforatum* population collected in 2007 and one *H. montanum* population collected in 1998. The seed material from seed banks and our field collections was collected according to ENSCONET guidelines (generally originating from at least 50 individuals; ENSCONET 2009). For the self‐collected material, seeds were sampled per mother plant, and an equal number of seeds from each mother were pooled for each temperature treatment (see Sections 2.3 and 2.4).

### 2.2 Germination Experiments

The seeds were cleaned from debris with an aspirator (Agriculex DB‐1, column seed cleaner CB115009) before sowing. For each population, we sowed a maximum of 200 seeds (depending on availability) 2 mm apart from each other on 8 petri dishes (25 seeds per petri dish, diameter 5.5 cm) filled with 1% agar (Sigma agar, lots SLBL4283V & SLBX7044). After 8 weeks of cold stratification at 4°C, we placed the petri dishes in growth chambers (Incubator numbers: LMS Cooled COLD 13234/20P4, MEAN 13233/20P4, WARM 13235/20P4, HOT 132636/20P4, United Kingdom), set to four temperature treatments. Daytime temperatures (16 h) were set to 16°C (cold), 20°C (medium), 24°C (warm) and 28°C (hot). The night time temperatures (8 h) were set to 10°C below the daytime temperature. Photoperiod in all treatments was 16/8 h light/dark. Each treatment was further divided into two replicates that were located on different shelves of the same growth chamber. The choice of temperature treatments was based on data on average summer temperatures at the trailing, core, and leading areas of the study species' ranges, as well as the predicted thermal conditions at the trailing edge in 2070, using data from WorldClim (Fick and Hijmans 2017).

### 2.3 Greenhouse Experiments

Seeds for the greenhouse experiments were cold‐stratified for 4 weeks at 4°C in dry paper bags. For each population, we sowed a maximum of 200 seeds (depending on availability) in 8 trays (25 seeds per tray) filled with sowing mixture (Kekkilä sowing mixture W HS R8017; KEK31116) covered by a thin layer (1–2 mm) of coarse sand. The trays were placed in greenhouse compartments with four temperature treatments identical to those used in the germination experiment, except that the night time temperature for the vegetative stage was changed to 8°C below the daytime temperature. Each treatment was further divided into two replicates placed in distinct greenhouse compartments. After the seeds had germinated, a maximum of 10 seedlings (depending on availability) were randomly chosen from each population and potted into individual 1 L pots filled with soil (Kekkilä Professional coarse potting mixture; KEK33933). The pots were placed on a water‐retaining rug on growing tables. The plants were watered by an automated watering system by soaking the rug underneath the pots. The watering schedule in each treatment was adjusted to keep the plants equally moist in all treatments. The plants were grown in the greenhouses from December 2021 to May 2022. At the end of March, the watering system broke leaving the *H. perforatum* plants dry for some days in replicate A. We accounted for this in the interpretation of the results. The plants were fertilized 6 weeks after sowing with a 0.075% solution of Kekkilä Turve Superex (NPK 12–5–27) and subsequently every 2 weeks with a 0.2% solution of the same fertilizer. To avoid any effects of differing conditions within the greenhouse compartment, the germination trays and pots were periodically rotated (dates of rotation: Dec 8, 2021, Dec 15, 2021, Dec 22, 2021, Jan 19, 2022, Jan 26, 2022, Feb 2, 2022, Mar 4, 2022, Apr 8, 2022).

### 2.4 Trait Measurements

During the germination experiments, we recorded the number of germinated seeds weekly over 4 weeks, based on seedlings having a radicle longer than 2 mm. Radicle length was defined as the distance from the radicle tip to the last occurrence of visible root hairs. Once a seed had been recorded as germinated, it was removed from the petri dish. In the fifth week, we performed cut‐tests to determine the viability of the remaining seeds, i.e., whether the seed had germinated during the last week of the experiment, or whether it was full, empty, moldy, or infested. At this stage, seedlings with a root radicle shorter than 2 mm were also scored as germinated. On the basis of this information, we determined the number of viable seeds (excluding empty and infested seeds) following the recommendations of the Millennium Seed Bank (Germination testing: procedures and evaluation. Millennium Seed Bank Partnership 2022). For estimating germination in further analyses, we included only populations where the proportion of seeds that germinated out of the total number of viable seeds was more than 5%. This was done to exclude accessions where low germination was likely due to incomplete dormancy release. From these measurements, we derived two traits for our analyses: germination proportion, defined as the proportion of viable seeds that germinated, and germination phenology, defined as the number of days from sowing to germination for seeds that emerged during the 4‐week monitoring period.

During the greenhouse experiments, we measured three traits: flowering phenology (the number of days from sowing to the onset of flowering), plant height (the length of the longest branch in cm), and flower abundance (the number of flowers produced by the end of the experiment, including withered and open flowers as well as full and empty seed capsules). Plant height and flower abundance were only measured for *H. montanum* and *H. perforatum* due to time limitations during data collection. The measured trait values across populations are given in Table S2.

### 2.5 Data Analyses

All data analyses were conducted in RStudio, version 2025.09.2 (Posit team 2025; R Core Team 2025).

#### 2.5.1 Measuring Range Position

**FIGURE 2:**
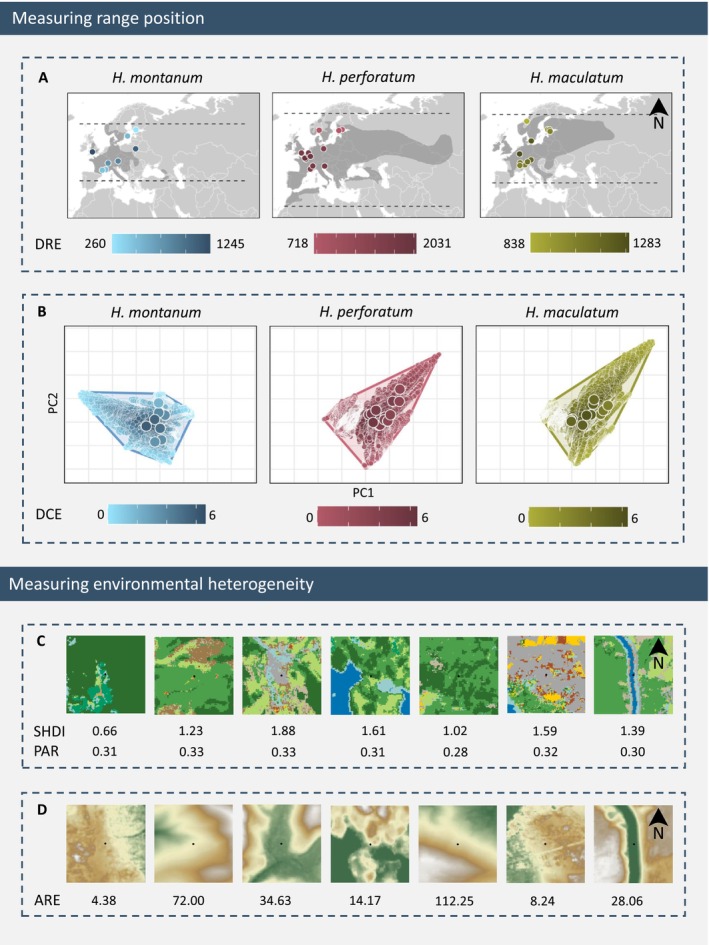
Illustration of metrics used for the population's range position and for environmental heterogeneity. Panel A illustrates distance to the southernmost or northernmost (dashed lines) range edge (DRE) with points in lighter colors indicating closer proximity to the range edge (in kilometers). Panel B illustrates distance to climatic edge (DCE) with larger points showing the study populations and smaller points all sites from which climatic parameters were measured across the species' range. Study populations in lighter colors were located closer to the species' climatic edge. Panels C and D illustrate the environmental heterogeneity metrics, showcased using examples of 500 × 500 m squares around seven populations of *H. montanum* . Squares in panel C show land cover types (Malinowski et al. 2020) from which Shannon diversity of land cover types (SHDI) and mean perimeter–area ratio of land cover patches (PAR) were calculated (square‐specific values of SHDI and PAR are shown in rows underneath each square). Squares in panel D show elevation (Copernicus Sentinel Data 2022) from which average roughness in elevation (ARE) values were calculated (square‐specific values of ARE are shown underneath each square).

We measured range position using two alternative metrics: (1) the geographic distance between the population and the edge of the species' range (DRE; Figure 2A), and (2) the climatic distance between the conditions at each source site and the average conditions across the range of the species (DCE; Figure 2B). To calculate these metrics, we digitized distribution maps from Hultén and Fries (1986) using QGIS (Transformation type: Thin Plate Spline; Resampling method: Lanczos; Coordinate system: Arctic Polar Stereographic EPSG:3995) (QGIS.org 2025). For DRE, we calculated the distance (in kilometers) from each study population's geographic location to the northernmost or southernmost range edge, whichever was located closer to the population (Figure 2A). To calculate DCE, we extracted and scaled (mean = 0, SD = 1) bioclimatic variables (WorldClim; Fick and Hijmans 2017) for each ~7 × 7 km (5 min) resolution cell that fell within the species' range and for each sampled population. We then conducted a Principal Component Analysis (PCA; Greenacre et al. 2022) for each species, and formed a convex hull around the first and second principal components to represent the species' climatic niche (Figure 2B; see loadings in Figure S3). The DCE metric was then calculated as the climatic distance from each study population's position to the nearest edge of the convex hull. Both metrics were finally scaled to zero mean and unit variance (mean = 0, SD = 1). The measured range position values across populations are given in Table S3.

#### 2.5.2 Measuring Environmental Heterogeneity

We measured environmental heterogeneity using three alternative metrics, two of which focus on different aspects of land cover heterogeneity, and one describing topographic heterogeneity. Land cover and topography were selected as the landscape features of interest because of their known impact on variation in microclimatic temperatures (Barry and Blanken 2016; Opedal et al. 2015), which in turn may select for thermal plasticity (Graae et al. 2018). More specifically, we calculated the Shannon diversity of land cover types (SHDI; Figure 2C), which represents compositional heterogeneity in land cover, mean of the perimeter–area ratio of land cover patches (PAR; Figure 2C), which represents configurational heterogeneity in land cover, and average roughness in elevation (ARE; Figure 2D), which represents topographic heterogeneity. To calculate SHDI and PAR, we used the function *calculate_lsm()* from the “landscapemetrics” R package (Hesselbarth et al. 2019) and to calculate ARE, we used the *sa*() function from the “geodiv” R package (Smith et al. 2021). We calculated all three metrics within a 500‐m square buffer (i.e., a square extending 500 m from the central point in all directions) around each study population. A square buffer was used to enable including whole grid cells in the buffer. Deciding the size of the square buffer was based on the estimated dispersal distance according to which the studied *Hypericum* species would be able to reach areas 500 m from the seed collection site within 100 years given yearly sexual reproduction (with a maximum dispersal distance of 5 m, Lososová et al. 2023). For SHDI and PAR, we used S2GLC 2017 land cover data (Malinowski et al. 2020) and for ARE we used EEA‐10 Copernicus DEM from year 2022 (Copernicus Sentinel Data 2022), both in 10‐m resolution. All three metrics were finally scaled to zero mean and unit variance (mean = 0, SD = 1). The measured environmental heterogeneity values across populations are given in Table S3.

#### 2.5.3 Quantifying the Effects of Range Position and Environmental Heterogeneity

##### 2.5.3.1 Estimating Thermal Plasticity

We fitted generalized linear mixed effects models (GLMMs) implemented with the *glmmTMB*() function in the “glmmTMB” R package (McGillycuddy et al. 2025) to model each trait as a population‐level linear function of temperature. We allowed populations to differ both in their mean deviation and in the slope of their reaction norms by including the interaction between population and treatment as a random effect (i.e., we fitted random‐regression models) (Arnold et al. 2019). To account for the nonindependence of individuals grown in the same greenhouse chamber or incubator, we included replicate ID as a random effect. We set Gaussian error distribution with an identity link function for all traits except germination, for which we set binomial error distribution with a logit link function. We treated temperature as a continuous variable, which we scaled to zero mean and unit variance (mean = 0, SD = 1) to facilitate model fitting. We evaluated normality and homoscedasticity of residuals by visually inspecting QQ‐plots and by plotting residuals against fitted values and statistically using Shapiro–Wilk and Breusch‐Pagan tests, and found minor deviations from normality and homoscedasticity (Figures S1 and S2). Flower abundance was square‐root‐transformed to better meet the assumptions. We extracted the random regression‐slope coefficients for each population, converted them to absolute values and used them as estimates of the magnitude of the population‐specific plastic response to temperature in the subsequent analyses. The measured plasticity values across populations are given in Table S3. To facilitate interpretation, we derived germination probabilities from the logit‐scale model estimates by applying the inverse logit function:

$$p=\frac{\exp\left(\eta \right)}{1+\exp\left(\eta \right)},$$

where *η* represents the linear predictor. We then calculated treatment effects as the difference between treatment probability and control probability. We used AIC (Akaike's Information Criterion) comparisons to assess statistical support for population‐specific responses to temperature by comparing models including only a random intercept to models including both a random intercept and a random slope for the population.

##### 2.5.3.2 Selecting Metrics With the Highest Explanatory Power for Variation in Thermal Plasticity

We fitted linear models (LMs) to test the effect of the two range position metrics (DRE and DCE) and the three environmental‐heterogeneity metrics (SHDI, PAR, and ARE) on thermal plasticity. For each trait, we fitted 12 models in total, to test all combinations of one range‐position and one environmental‐heterogeneity variable, as well as their interaction. Thus, each model included a maximum of two explanatory variables and their interaction. We also tested whether accounting for non‐independence between the populations of the same species improved the models but found no qualitative change in the results (see Tables S4 and S5). We computed Spearman correlations among explanatory variables using the *cor_test*() function in the ‘rstatix’ R package (Kassambara 2025), and all were below 0.7, indicating no problematic multicollinearity (Dormann et al. 2013). We ranked the models based on AIC values, and for each trait selected the model with the lowest AIC value (ΔAIC > 2; Burnham and Anderson 2004; Symonds and Moussalli 2011). If the highest‐ranked model included an interaction, this parameter was also incorporated into the final models. If there were no detectable differences between the models, we selected the one with the lowest AIC value for further investigation and assessed case by case whether any of the competing models provided additional insight. We evaluated normality and homoscedasticity of residuals by visually inspecting QQ‐plots and by plotting residuals against fitted values and statistically using Shapiro–Wilk and Breusch‐Pagan tests, and found no major deviations from normality and homoscedasticity (Figures S4 and S5).

##### 2.5.3.3 Analyzing the Effect of Range Position and Environmental Heterogeneity

To analyze the effect of range position and environmental heterogeneity on among‐population thermal plasticity, we assessed the parameter estimates of the models selected in the previous step (Section 2.5.3.2). Additionally, we examined the amount of variance explained by each term by calculating their relative importances with the *calc.relimp*() function from the “relaimpo” R package (Groemping 2005). Predictions were produced using the *ggpredict*() function in the “ggeffects” R package (Lüdecke 2018).

## 3 Results

### 3.1 Thermal Plasticity and Its Variation Among Populations

The log‐odds of a *H. perforatum* seed germinating declined, on average, 0.08 logit units per 1°C (*p* < 0.001), equivalent to a 1.3 percentage point decrease in germination probability per 1°C temperature increase. In addition to this, for all three species, the timing of germination and flowering advanced, on average, with increasing temperature (Table 1). Specifically, the timing of germination advanced 0.5 days for *H. montanum* (*p* < 0.05), and 0.7 days for *H. perforatum* (*p* < 0.001) and *H. maculatum* (*p* < 0.001) per 1°C. Moreover, *H. montanum* plants flowered 3.6 days (*p* < 0.001), *H. perforatum* 4 days (*p* < 0.001), and *H. maculatum* 3.5 days (*p* < 0.001) earlier per 1°C (Table 1). For the remaining species‐trait combinations, we failed to detect thermal responses across populations. The average thermal responses were rather similar across species for all traits (Table 1).

**TABLE 1:** Parameter estimates from models describing plastic responses to temperature treatments.

Trait	Species	Term	Estimate	SE	*t*	*p*	SD_pop_	Min_pop_	Max_pop_	ΔAIC
Germination probability (log‐odds/°C)	*H. montanum*	(Intercept)	−1.489	0.151	−11.324	< 0.001				
		Treatment	−0.010	0.048	−0.205	0.838	0.078	−0.107	0.087	6.47
	*H. perforatum*	(Intercept)	1.307	0.181	−2.318	< 0.05				
		Treatment	−0.078	0.021	−3.749	< 0.001	0.053	−0.178	0.032	23.72
	*H. maculatum*	(Intercept)	1.162	0.212	1.139	0.255				
		Treatment	−0.042	0.038	−1.110	0.267	0.052	−0.141	0.034	13.05
Germination phenology (days/°C)	*H. montanum*	(Intercept)	24.879	1.536	8.225	< 0.001				
		Treatment	−0.548	0.239	−2.290	< 0.05	0.301	−0.831	0.048	0.41
	*H. perforatum*	(Intercept)	32.721	0.747	26.190	< 0.001				
		Treatment	−0.650	0.152	−4.268	< 0.001	0.163	−0.948	−0.394	0.70
	*H. maculatum*	(Intercept)	32.045	1.027	17.225	< 0.001				
		Treatment	−0.656	0.167	−3.939	< 0.001	0.061	−0.722	−0.499	2.80
Flowering phenology (days/°C)	*H. montanum*	(Intercept)	199.452	4.101	29.950	< 0.001				
		Treatment	−3.614	0.883	−4.091	< 0.001	0.598	−4.382	−2.819	0.80
	*H. perforatum*	(Intercept)	209.075	3.008	41.557	< 0.001				
		Treatment	−3.992	0.592	−6.748	< 0.001	1.190	−5.676	−1.883	20.7
	*H. maculatum*	(Intercept)	192.556	2.798	40.328	< 0.001				
		Treatment	−3.493	0.805	−4.339	< 0.001	1.404	−5.447	−1.944	14.2
Plant height (cm/°C)	*H. montanum*	(Intercept)	102.402	6.622	9.814	< 0.001				
		Treatment	−1.765	1.250	−1.412	0.158	1.602	−4.092	−0.148	15.8
	*H. perforatum*	(Intercept)	47.673	2.536	24.896	< 0.001				
		Treatment	0.734	0.506	1.452	0.147	0.883	−0.74	2.57	33.5
Flower abundance (flowers/°C)	*H. montanum*	(Intercept)	277.689	3.633	4.258	< 0.001				
		Treatment	−0.162	0.203	−0.899	0.369	0.629	−1.464	0.001	28.6
	*H. perforatum*	(Intercept)	84.213	3.165	4.950	< 0.001				
		Treatment	−0.000	0.051	−0.078	0.938	0.133	−0.350	0.196	29.1

*Note:* The estimates and standard errors shown here have been back‐transformed to the original scale (unit/°C). SE = standard error in the average thermal responses of the species; SD_pop_ = standard deviation of the population‐specific thermal responses; Min_pop_ = minimum of the population‐specific thermal responses; Max_pop_ = maximum of the population‐specific thermal responses; ΔAIC = difference in the AIC (Akaike's Information Criterion) value between the model including both random intercept and slope and only random intercept for population, with values greater than 2 indicating support for the random‐slope component.

**FIGURE 3:**
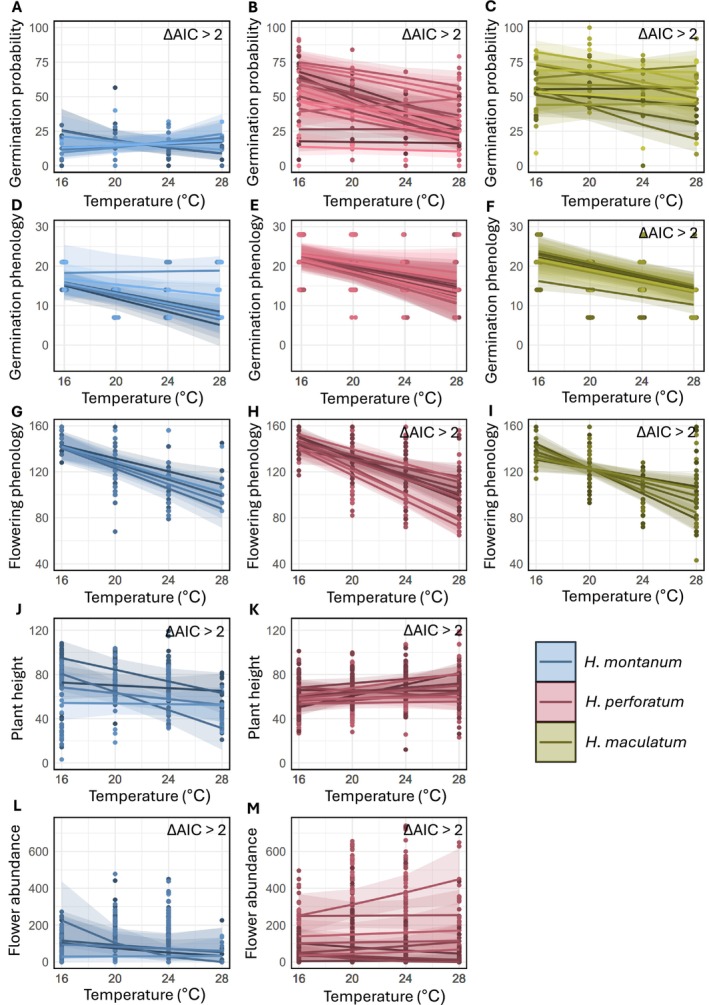
Predicted population‐specific effects of temperature (°C) on the five studied traits. A–C germination probability (converted to percentages); D–F germination phenology (number of days after sowing); and G–I flowering phenology (number of days after sowing) for *H. montanum* (blue), *H. perforatum* (red), and *H. maculatum* (green). J,K plant height (cm) and L,M number of flowers for *H. montanum* and *H. perforatum* . The estimates and standard errors shown here have been back‐transformed to the original scale. Points show the measured trait values in each temperature treatment, and lines show the predicted trait values across temperature treatments and their 95% confidence intervals. Points and lines for different populations are shown within each plot with different shades of color. ΔAIC > 2 indicates statistical support for among‐populational differences in thermal responses.

All species exhibited population‐specific responses of germination probability, and *H. montanum* and *H. perforatum* of plant height and flower abundance (for which we had data only for these two species), as indicated by support for the models including both random intercepts and slopes for population (Table 1). For germination probability, the population‐specific responses varied from a 0.11 log‐odds reduction per 1°C to a 0.09 log‐odds increase per 1°C (Table 1; Figure 3A–C), equivalent to a 6.3 percentage point reduction per 1°C to an 8.1 percentage point increase per 1°C. For plant height, the population‐specific responses varied from a 4.1 cm reduction per 1°C to a 2.58 cm increase per 1°C (Table 1; Figure 3J,K), and for flower abundance from 1.5 flowers decrease per 1°C to 0.2 flowers increase per 1°C (Table 1; Figure 3L,M). The thermal responses in germination phenology were more uniform among populations, varying from an advance of 1 day/°C to a delay of 0.05 days/°C (Table 1; Figure 3 D–F), with statistical support for variation in germination phenology only for *H. maculatum* . For flowering phenology, the population‐specific responses ranged from a 5.7 to a 1.9‐day advance per 1°C (Table 1; Figure 3 G–I), providing strong evidence of intraspecific variation in thermal responses of *H. maculatum* and *H. perforatum* . For all traits but flowering phenology, intraspecific variation in thermal responses was greatest in *H. montanum* .

### 3.2 Effect of Range Position and Environmental Heterogeneity on Thermal Plasticity

In the highest‐ranked (lowest AIC) models, distance to range edge (DRE) represented range position for plasticity in plant height and flower abundance, while distance to the climatic edge (DCE) represented range position for plasticity in germination probability, germination phenology, and flowering phenology (Table 2). Similarly, environmental heterogeneity was best captured by the mean perimeter–area ratio of land cover patches (PAR) for plant height, the Shannon diversity of land cover types (SHDI) for germination probability, and average roughness in elevation (ARE) for germination phenology, flowering phenology, and flower abundance (Table 2). The pairwise interaction between range position and environmental heterogeneity was retained in the highest‐ranked models for plasticity in germination phenology, plant height, and flower abundance (Table 2). The correlations between the range position and environmental heterogeneity metrics, as well as within them, were relatively low: the highest between‐correlation emerged between distance to range edge (DRE) and average roughness in elevation (ARE) (ρ = −0.35; Table S6), while the highest within‐correlation emerged between distance to range edge (DRE) and distance to climatic edge (DCE) (ρ = −0.4).

**TABLE 2:** Model selection results for models testing the effect of range position and environmental heterogeneity on trait plasticity.

Range position metric used	Environmental heterogeneity metric used	Interaction included	Plasticity in germination probability	Plasticity in germination phenology	Plasticity in flowering phenology	Plasticity in plant height	Plasticity in flower abundance
			ΔAIC	ΔAIC	ΔAIC	ΔAIC	ΔAIC
DRE	SHDI	No	1.4	5.0	6.3	10.2	10.5
DRE	SHDI	Yes	1.6	3.8	6.9	12.0	11.7
DRE	PAR	No	2.3	5.0	3.2	9.1	11.4
DRE	PAR	Yes	1.7	4.4	3.1	0.0	7.8
DRE	ARE	No	2.3	5.1	0.8	10.3	11.7
DRE	ARE	Yes	4.0	5.4	2.7	0.3	0.0
DCE	SHDI	No	0.0	5.7	6.0	12.0	11.2
DCE	SHDI	Yes	2.0	3.9	7.7	12.5	13.2
DCE	PAR	No	0.5	5.6	3.6	10.3	11.6
DCE	PAR	Yes	1.9	7.4	5.4	12.2	13.2
DCE	ARE	No	1.3	5.7	0.0	11.9	12.1
DCE	ARE	Yes	3.2	0.0	1.3	12.5	12.7

*Note:* Each tested model included one variable from each group, with one version of the model including their interaction.

Abbreviations: ΔAIC, difference in the AIC (Akaike's Information Criterion) value between the model compared to the highest‐ranked model (ΔAIC, 0.0); ARE, average roughness in topography; DCE, distance to climatic edge; DRE, distance to range edge; PAR, mean perimeter–area ratio of land cover patches; SHDI, Shannon diversity of land cover types.

For plasticity in germination phenology and flower abundance, the highest‐ranked model was supported over all alternative candidate models (Table 2). For the other studied traits, there was also support for additional models (6, 2, and 1 additional models for plasticity in germination probability, flowering phenology and plant height, respectively; Table 2). Across these alternative models, the effects of range position and environmental heterogeneity were largely consistent (Table S7): for plasticity in flowering phenology, both effect sizes and directions remained unchanged; for plant height, support for the effect of environmental heterogeneity increased to moderate (*p* < 0.1); and for germination, the effect of range position decreased when measured using distance to range edge (DRE) compared to using distance to climatic edge (DCE), as included in the highest‐ranked model.

**FIGURE 4:**
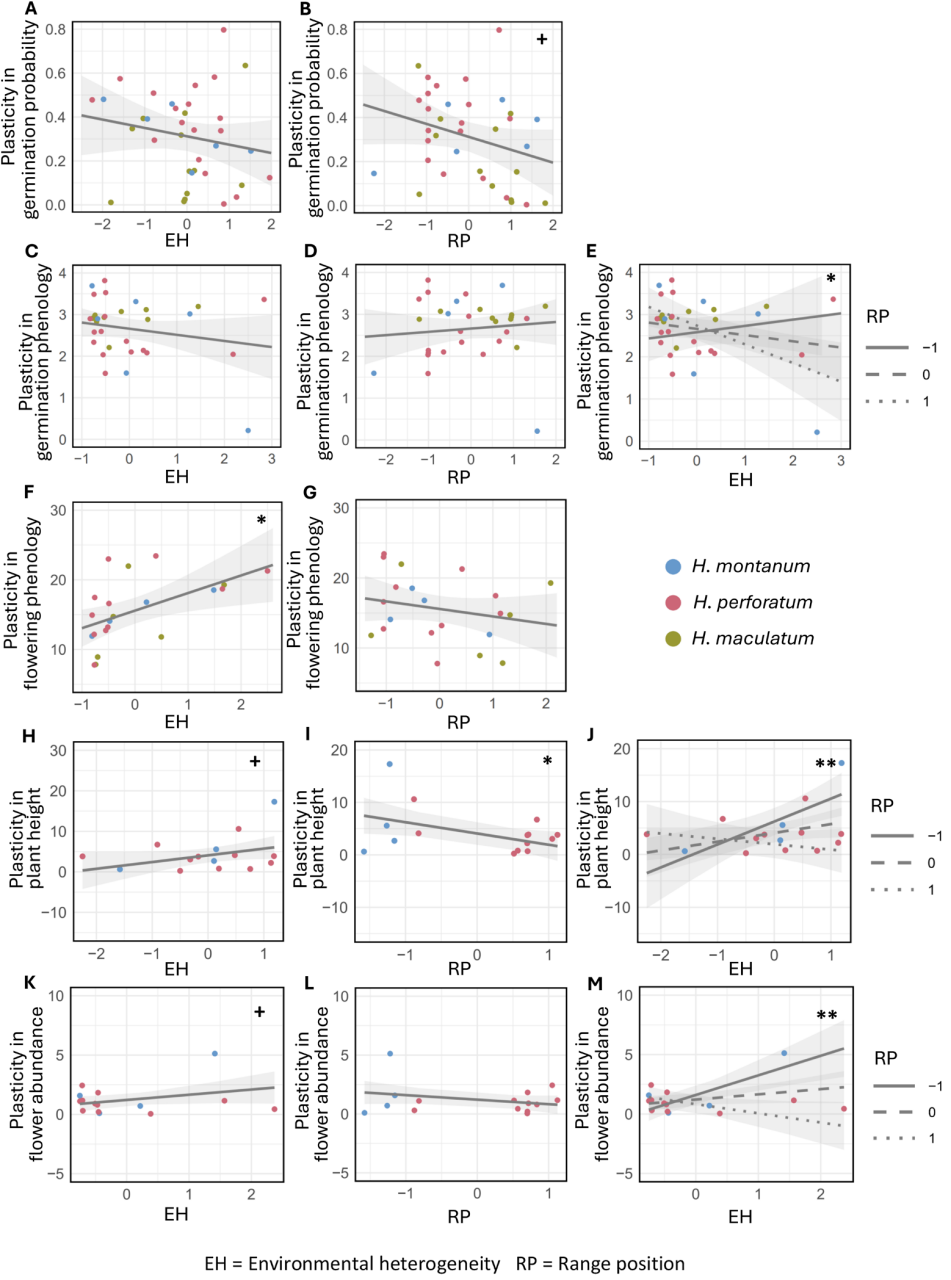
Predictions from the highest‐ranked models testing the effect of range position (RP) and environmental heterogeneity (EH) on trait plasticity, with population‐specific plasticity values in blue ( *H. montanum* ), red ( *H. perforatum* ), and green ( *H. maculatum* ). For RP and EH metrics, value −1 indicates the lowest distance to edge/lowest amount of environmental heterogeneity and value 1 the greatest distance to edge/highest amount of environmental heterogeneity. A,B germination probability; C–E germination phenology; and F,G flowering phenology; H–J plant height and K–M flower abundance. Statistically significant *p*‐values are shown with *p* < 0.01 = **; *p* < 0.05 = *; *p* < 0.1 = +.

The highest‐ranked models provided moderate evidence for independent effects of range position and environmental heterogeneity on plasticity in flowering phenology and plant height (Table 3; Figure 4). Populations located farther from the edge were less plastic in height (Est. = −2.15; *p* < 0.05; Table 3), while populations located in more heterogeneous environments were more plastic in flowering phenology (Est. = 2.52; *p* < 0.05; Table 3). Furthermore, plasticity in germination probability tended to vary with range position, and plasticity in plant height and flower abundance tended to vary with environmental heterogeneity. Populations located farther from the edge were less plastic in germination probability (Est. = −0.06; *p* < 0.1; Table 3), while populations in more heterogeneous environments were more plastic in plant height (Est. = 1.67; *p* < 0.1) and flower abundance (Est. = 0.44; *p* < 0.1).

**TABLE 3:** Parameter estimates from the highest‐ranked models testing the effect of range position (RP) and environmental heterogeneity (EH) on trait plasticity. SE = standard error.

Trait plasticity	Term	Estimate	SE	*t*	*p*
Plasticity in germination probability	Intercept	0.312	0.033	9.580	< 0.001
	EH	−0.038	0.033	−1.151	0.258
	RP	−0.059	0.033	−1.770	< 0.1
Plasticity in germination phenology	Intercept	2.660	0.115	23.125	< 0.001
	EH	−0.147	0.119	−1.232	0.228
	RP	0.078	0.120	0.652	0.520
	EH:RP	−0.296	0.107	−2.768	< 0.05
Plasticity in flowering phenology	Intercept	15.578	0.883	17.636	< 0.001
	EH	2.524	0.906	2.786	< 0.05
	RP	−1.071	0.906	−1.182	0.253
Plasticity in plant height	Intercept	4.075	0.804	5.070	< 0.001
	EH	1.660	0.844	1.968	< 0.1
	RP	−2.146	0.852	−2.520	< 0.05
	EH:RP	−2.679	0.772	−3.471	< 0.01
Plasticity in flower abundance	Intercept	1.216	0.228	5.333	< 0.001
	EH	0.439	0.240	1.828	< 0.1
	RP	−0.385	0.240	−1.603	0.137
	EH:RP	−1.212	0.300	−4.046	< 0.01

For those traits for which the highest‐ranked model included an interaction between range position and environmental heterogeneity metrics (germination phenology, plant height, and flower abundance) the interaction term was negative (Est. = −0.29; *p* < 0.05, Est. = −2.68; *p* < 0.05, Est. = −1.21; *p* < 0.05 respectively; Table 3). This means that there was a tendency for populations closer to the edge to have greater plasticity at greater environmental heterogeneity, and *vice versa* in populations toward the core areas (Figure 4).

**FIGURE 5:**
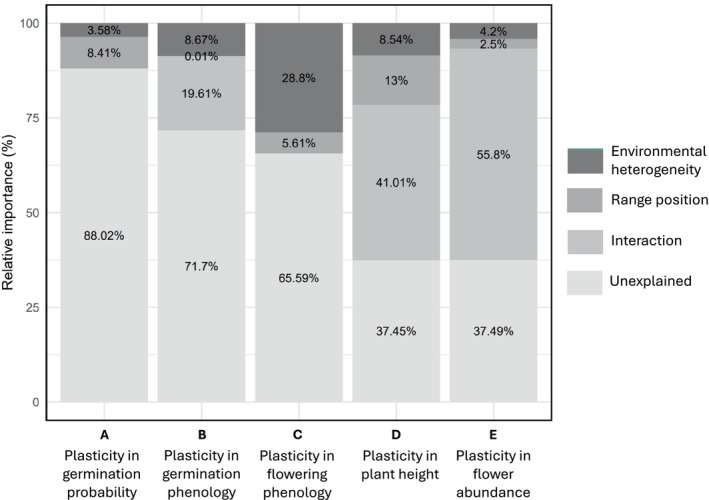
Variance partitioning of models testing the effects of range position and environmental heterogeneity on trait plasticity. Stacked bars represent the proportion of total variance in plasticity (R^2^) attributable to each predictor, in addition to unexplained variance.

We found differences among traits in how much variation in plasticity was explained by the predictors. The percentage of variation in plasticity explained was lowest for germination probability (12%; Figure 5), and highest for plant height (62.6%) and flower abundance (62.6%). Furthermore, we found differences in which of the predictors explained most of the plasticity variation. For traits for which models included an interaction between range position and environmental heterogeneity, the interaction effect explained the majority of the variation. The percentage of variance explained by the interaction term was particularly high in the models for plant height (41.0%) and flower abundance (55.8%), and moderate for germination phenology (19.6%). For the traits for which the highest‐ranking models did not include an interaction term, range position was more important for germination probability while environmental heterogeneity was more important for flowering phenology.

## 4 Discussion

### 4.1 Thermal Plasticity and Its Variation Among Populations

Our results revealed detectable thermal plasticity in germination and flowering phenology for all three species, and for *H. perforatum* in germination probability. Consistent with other studies, warmer temperatures led to earlier germination and flowering (Collins et al. 2025; de Villemereuil et al. 2018; Haggerty and Galloway 2011), and to a reduced probability of germination (Vázquez‐Ramírez and Venn 2025). For the remaining traits (plant height and flower abundance) we did not detect thermal responses across populations. These patterns support the idea that plasticity is often trait‐specific (Arnold et al. 2022; Hällfors et al. 2025; Ren et al. 2020), with phenological traits, in particular, being highly sensitive to temperature (Cleland et al. 2007; Menzel et al. 2020; Roslin et al. 2021).

While we found little variation in average thermal responses among the species for most of the traits, we did detect variation among conspecific populations for the majority of them. For germination probability, plant height, and flower abundance, conspecific populations of all tested species (for germination probability all three species and for plant height and flower abundance *H. montanum* and *H. perforatum* ) differed in their thermal responses, while for flowering phenology, such variation was evident for *H. perforatum* and *H. maculatum* , and for germination phenology for *H. maculatum* . For all of these species‐trait combinations, the populations varied both in the magnitude and direction of the thermal response (Table 1; Figure 3). Interestingly, in all traits but flowering phenology, we found most variation among the populations of *H. montanum* , potentially indicating stronger local adaptation induced by the more sparse occurrence, and thus more limited gene flow between the populations of the species.

Overall, our findings align with a recent meta‐analysis indicating that intraspecific variation in plasticity is common in plants (Matesanz and Ramírez‐Valiente 2019) and highlight the importance of accounting not only for the average responses of the species, but also for intraspecific variation. High intraspecific variation in plasticity can allow some populations to respond adaptively to climate change (Walter et al. 2023, 2026), rendering species‐level assumptions prone to erroneous predictions. Thus, these results suggest that the three *Hypericum* species possess genetic variation for diverse plastic responses that could enable some of the populations to adjust more efficiently to environmental changes induced by climate change.

Because the tested temperatures covered a wide range of current and potential future growing season temperatures across the species ranges, we consider it unlikely that a wider range of temperatures would have revealed substantial additional plasticity. However, exploring potential nonlinear responses could uncover plasticity that remained undetected in our analysis (Arnold et al. 2022). To avoid overcomplicated models, we extracted the slope of the linear response to then use for our subsequent test of the roles of range position and environmental heterogeneity. Thus, examining non‐linear effects was beyond the scope of this particular study. Future studies should, however, account for potential nonlinear responses to temperature to investigate the extent of thermal plasticity.

### 4.2 Relationship Between Thermal Plasticity and Range Position

We found moderate evidence that plasticity in plant height and germination probability declined with increasing distance to the range edge (Table 3; Figure 4). Populations located closer to the edge of the species' range were more plastic in plant height and germination probability than those located closer to the core. For the other three traits (germination phenology, flowering phenology and flower abundance) range position had no detectable independent effect. As outlined in the introduction, phenotypic plasticity is often hypothesized to vary within species ranges either by peripheral populations showing lower plasticity (Mägi et al. 2011), or greater plasticity (Zettlemoyer and Peterson 2021), in comparison to core populations. Our results concerning thermal plasticity in plant height and germination probability, together with e.g., Molina‐Montenegro and Naya (2012) and Lázaro‐Nogal et al. (2015), provide support for the latter hypothesis, indicating that plasticity can, in some cases, be higher for peripheral populations.

The trend of increasing plasticity toward the periphery could be connected to patterns in species' abundances and, potentially simultaneously, to gradients in environmental heterogeneity aligning with range position. Although empirical evidence remains contested (e.g., Panter et al. 2025), the abundance of species is often assumed to be highest at the core of a species' range and decline toward the periphery, due to a similar pattern in environmental suitability (Brown 1984). Assuming this applies, in less abundant peripheral populations, greater plasticity may evolve as a consequence of directional selection maintained by maladaptive gene flow from the more abundant core populations (Chevin and Lande 2011). Additionally, greater seasonal variation in climatic conditions at the poleward edges could favor the selection of plasticity in peripheral populations (Addo‐Bediako et al. 2000). Because we measured neither the amount of genetic variation within the sampled populations nor seasonal variation in climatic conditions, we can, however, only speculate on whether these mechanisms relate to the patterns we observed.

### 4.3 Relationship Between Thermal Plasticity and Environmental Heterogeneity

Plasticity in flowering phenology, plant height and flower abundance tended to increase with environmental heterogeneity, although with variable statistical support (Table 3; Figure 4). Thus, populations located in more heterogeneous landscapes responded more plastically to temperature increase by, for instance, advancing flowering more than populations located in more homogeneous landscapes. This could be a result of the offspring of plant individuals in the populations located in the more heterogeneous landscapes needing to germinate in contrasting environmental patches compared to the parental plant, which in turn may result in selection for plasticity (Alpert and Simms 2002). In other words, greater thermal plasticity may have evolved to allow populations to inhabit such variable environments (Graae et al. 2018). For the remaining two traits—germination probability and germination phenology—environmental heterogeneity did not have a detectable independent effect.

The results for plasticity in flowering phenology, plant height and flower abundance are in line with previous theoretical work indicating that environmental heterogeneity can favor phenotypic plasticity (Botero et al. 2015; Scheiner 2013; Sultan and Spencer 2002; Tufto 2000). However, most previous empirical studies testing the effect of environmental heterogeneity on plasticity have focused on temporal environmental heterogeneity rather than spatial environmental heterogeneity (e.g., Gianoli 2004; Gianoli and González‐Teuber 2005; Lázaro‐Nogal et al. 2015). In the rare cases where the effect of spatial environmental heterogeneity has been tested, plasticity has been found to be driven by the spatial scale rather than the magnitude of environmental heterogeneity surrounding the population (Baythavong 2011; de la Mata et al. 2022). Our study thus represents one of the first empirical studies finding signatures of greater plasticity in more spatially heterogeneous environments.

### 4.4 Interactive Effect of Range Position and Environmental Heterogeneity on Thermal Plasticity

We found moderate evidence for range position and environmental heterogeneity interactively affecting plasticity in all of the three traits where the highest‐ranked model included the interaction, i.e., plasticity in germination phenology, plant height, and flower abundance. Furthermore, we found the direction of the interaction to be consistent across the three traits: an increase in environmental heterogeneity was linked to an increase in plasticity in peripheral populations, but to a decrease in plasticity in core populations. This interaction was the only pathway through which range position and environmental heterogeneity affected plasticity in germination phenology. For plasticity in plant height, both range position and environmental heterogeneity also had an independent effect, and for plasticity in flower abundance, environmental heterogeneity additionally had an independent effect.

Based on previous hypotheses about how range position and environmental heterogeneity may affect plasticity, we formulated two alternative hypotheses (see *Introduction*) for the direction of the interaction. First, we hypothesized that the positive impact of increased environmental heterogeneity on plasticity may be dampened in peripheral populations as, e.g., lower genetic variation may restrict the evolutionary potential for increasing phenotypic plasticity (Arnaud‐Haond et al. 2006; Ellstrand and Elam 1993). Second, we hypothesized that the positive impact of increased environmental heterogeneity on plasticity may be further enhanced in peripheral populations if, e.g., directional selection, maintained by maladaptive gene flow from central populations (Chevin and Lande 2011), selects for the evolution of higher plasticity.

While the exacerbating impact of environmental heterogeneity toward the peripheral populations was in line with our second hypothesis, the abating effect toward the core was not. Some studies have, however, suggested that selection may work against plasticity in highly variable environments due to high levels of environmental stress caused by the varying conditions (Mägi et al. 2011). In such environments, the energetic costs of maintaining flexibility may outweigh its benefits, making a less plastic, more robust phenotype a more successful strategy (Schneider 2022). In addition to this, increased environmental heterogeneity may lead to selection against phenotypic plasticity if environmental heterogeneity is too unpredictable, and the phenotype developed through the plastic response does not match the optimal phenotype (Leung et al. 2020). In the case of higher abundance at the core areas of species' ranges, core populations may have higher connectivity and gene flow, which could help maintain genetic variation for selection to act upon (Eckert et al. 2008), potentially making core populations particularly capable of responding to those high levels of environmental stress or unpredictable environmental conditions. However, because we did not collect data on genetic variation within populations, species‐specific environmental stress conditions, or on the predictability of the environmental heterogeneity, we can only speculate whether these mechanisms could cause a negative effect of environmental heterogeneity on plasticity in core populations. Nevertheless, the contrasting associations between environmental heterogeneity and plasticity across the species' ranges underscore the complexity of variation in thermal plasticity across species ranges, and accentuates the substantial knowledge gaps that still remain unresolved.

### 4.5 To What Extent Was Plasticity Predicted?

Our results revealed substantial differences among traits in how much variation in plasticity could be explained by range position, environmental heterogeneity, and their interaction. The explanatory power was particularly high for plasticity in plant height (62.6%) and flower abundance (62.5%), for which the interaction between range position and environmental heterogeneity accounted for most of the explained variation (41% and 55.8%, respectively), highlighting its crucial role in predicting plasticity in those traits. In contrast, for the other three traits, a large proportion of the variation (65.6%–88%) remained unexplained.

Similar trait‐specific patterns have been reported, for instance, in a recent global meta‐analysis, where an effect of latitude was found only for certain traits when assessing plasticity across many species and studies (Dobson and Zarnetske 2025). Furthermore, Manenti et al. (2017) showed that environmental heterogeneity had weak and trait‐specific effects on plasticity in three sympatric *Drosophila* species. We can speculate that the trait‐specific effects of range position and environmental heterogeneity on plasticity are related to how closely the traits are linked to fitness, although they may also indicate that additional factors influencing plasticity were not included in our models. For instance, it is possible that some of the unexplained variation could arise from stochastic processes such as genetic drift (Lenormand et al. 2009), which are not straightforward to incorporate into analyses on intraspecific plasticity using designs such as ours. Furthermore, for some traits, environmental heterogeneity that occurs at a spatial scale smaller than in which it was measured here (10 × 10 m) may determine the degree of plasticity. However, such data are rarely available, especially for spatial extents as large as ours.

Finally, it is possible that some of the unexplained variation originates from intrinsic differences among the tested species. Due to the relatively low number of studied populations, we were not able to assess whether the effect of range position and environmental heterogeneity differs among the species. However, it is possible that in the more sparsely occurring *H. montanum* , reduced connectivity among populations may lead to lower gene flow and stronger local adaptation, potentially resulting in more pronounced differences in thermal plasticity between peripheral and core populations compared to the more widespread *H. perforatum* and *H. maculatum* . Additionally, the fact that *H. montanum* and *H. maculatum* are strict sexual reproducers, while *H. perforatum* is facultatively apomictic, could lead to differences in the genetic constitution of the species (Schoen and Brown 1991) and thus in how the populations of the species respond to selection, specifically rendering *H. montanum* and *H. maculatum* populations more sensitive to selection. The fact that the percentage of variation explained was particularly high for the two traits for which we had data from only two of the three species may point to species‐specific differences: incorporating the third species may have introduced variation that we were not able to explain. Future studies should investigate whether thermal responses differ between common versus rarer species. Such work could reveal important information about distinct drivers of intraspecific patterns among species and could be especially useful for informing conservation decisions.

### 4.6 Does Range Position Constitute a Proxy for Local‐Scale Environmental Heterogeneity?

Our results revealed low correlations between the two range‐position and three environmental‐heterogeneity metrics. These results suggest that, at least in our study system, and in terms of spatial environmental heterogeneity, range position does not constitute a functional proxy for local‐scale environmental heterogeneity. This result gives support for earlier studies suggesting that local‐scale environmental heterogeneity may disrupt latitudinal patterns in environmental heterogeneity (Manenti et al. 2017; Noer et al. 2022). However, when comparing the relative importances of range position and local‐scale environmental heterogeneity, we did not find clear evidence, across traits, that one would be more important than the other and thus that local‐scale environmental heterogeneity would override the impact of range position. In fact, their substantial interactive role in explaining variation in plasticity for two of the traits suggests that range position and environmental heterogeneity may best predict plasticity when they are both accounted for.

## 5 Conclusions and Future Directions

Phenotypic plasticity is expected to play a key role in the performance of populations under climate change. Our study demonstrates that while thermal phenotypic plasticity in the studied traits was relatively similar among the three *Hypericum* species examined, it varied among the populations of the species. Such variation may result in differences in the ability of the populations, and subsequently the species, to cope with the impacts of climate change. In future studies, it will be important to connect data such as ours with data on observed and expected changes in thermal environments to better understand and predict historical and future trajectories for populations and how this varies across the species' range.

Importantly, our results provide interesting indications that both a population's position within the species' range and the local‐scale environmental heterogeneity surrounding the population may simultaneously, and also perhaps mechanistically interactively, be connected to plasticity. More specifically, our study indicates that plasticity may vary along several interacting environmental axes, and particularly that higher plasticity in edge populations in heterogeneous environments can confer them with an advantage to cope with changes in climatic conditions through plastic responses.

Overall, our results highlight the need for integrated research that jointly assesses the roles of populations' position within range and environmental heterogeneity in determining the degree of phenotypic plasticity. This could potentially help address the inconsistencies that remain especially among the studies assessing the effect of range position. Moreover, the causal mechanism of why the populations' position within range is connected to plasticity should be investigated further. This would improve our understanding of geographic patterns in the plasticity of species and populations, their ability to respond to environmental change via adaptive plastic responses, and ultimately help predict the future viability of biodiversity under continued and intensifying environmental change.

## Author Contributions

**S. H. M. Koivusaari:** conceptualization (lead), data curation (lead), formal analysis (lead), funding acquisition (equal), investigation (equal), methodology (lead), project administration (lead), software (lead), validation (lead), writing – original draft (lead), writing – review and editing (lead). **M. H. Hällfors:** conceptualization (equal), data curation (equal), formal analysis (supporting), funding acquisition (equal), investigation (lead), methodology (equal), project administration (equal), resources (supporting), supervision (lead), validation (supporting), writing – original draft (equal), writing – review and editing (lead). **J. Hjort:** conceptualization (supporting), supervision (equal), writing – review and editing (supporting). **M.‐T. Hyvärinen:** funding acquisition (equal), investigation (supporting), project administration (supporting), supervision (equal), writing – review and editing (supporting). **M. Levo:** investigation (supporting), writing – review and editing (supporting). **M. Luoto:** supervision (supporting), writing – review and editing (supporting). **C. Møller:** writing – review and editing (equal). **Ø. H. Opedal:** investigation (supporting), methodology (equal), software (supporting), supervision (equal), writing – review and editing (equal). **L. Pietikäinen:** data curation (equal), investigation (lead), writing – review and editing (supporting). **A. Romero‐Bravo:** investigation (supporting), writing – review and editing (equal). **A. L. K. Mattila:** conceptualization (equal), data curation (lead), formal analysis (supporting), funding acquisition (supporting), investigation (lead), methodology (equal), project administration (equal), resources (supporting), supervision (lead), validation (supporting), writing – original draft (equal), writing – review and editing (lead).

## Funding

This work was supported by Research Council of Finland, 324555, 330739, 360742. Koneen Säätiö. Jane ja Aatos Erkon Säätiö. Societas pro Fauna et Flora Fennica. Waldemar von Frenckells Stiftelse. Nordenskiöld‐samfundet.

## Conflicts of Interest

The authors declare no conflicts of interest.

## Supporting information


**Figure S1:** QQ‐plots of the models testing the effect of temperature on the traits. These were used to assess normality of residuals. A–C Germination probability; D–F germination phenology; and G–I flowering phenology for *H. montanum* (blue), *H. perforatum* (red), and *H. maculatum* (green), respectively J–K plant height and L–M flower abundance for *H. montanum* and *H. perforatum* . The normality of the residuals was additionally tested using Shapiro–Wilk test, where the *p*‐values for *H. montanum* , *H. perforatum* , and *H. maculatum* were 0.07, 0.11, and 0.31 for germination probability; 0.06, 0.00, and 0.00 for germination phenology; 0.03, 0.04, 0.00 for flowering phenology; 0.00, 0.45 for plant height; 0.00, 0.00 for flower abundance.
**Figure S2:** Residuals versus fitted values for the models testing the effect of temperature on the traits. These were used to assess homoscedasticity of residuals. A–C Germination probability, D–F germination phenology; and G–I flowering phenology for *H. montanum* (blue), *H. perforatum* (red), and *H. maculatum* (green), respectively J,K plant height and L,M flower abundance for *H. montanum* and *H. perforatum* . The homoscedasticity of the residuals was additionally tested using Breusch‐Bagan test, where the *p*‐values for *H. montanum* , *H. perforatum* and *H. maculatum* were 0.00, 0.00, 0.00 for germination probability; 0.00, 0.00, 0.91 for germination phenology; 0.02, 0.00, 0.00 for flowering phenology; 0.00, 0.45 for plant height; 0.06, 0.00 for flower abundance.
**Figure S3:** Loadings of bioclimatic variables on Principal Component (PC) 1 and 2. Loadings quantify the contribution of each variable to a principal component. Descriptions of bioclimatic variables can be found at https://www.worldclim.org/data/bioclim.html.
**Figure S4:** QQ‐plots for the models testing the effect of position within range and environmental heterogeneity on trait plasticity. These were used to assess normality of residuals. The normality of the residuals was additionally tested using Shapiro–Wilk test, where the *p*‐values were 0.42 for plasticity in germination probability; 0.38 for plasticity in germination phenology; 0.30 for plasticity in flowering phenology; 0.87 for plasticity in plant height; 0.87 for plasticity in flower abundance.
**Figure S5:** Residuals versus fitted values based on the models testing the effects of position within range and environmental heterogeneity on trait plasticity. These were used to assess homoscedasticity of residuals. The homoscedasticity of the residuals was additionally tested using Breusch‐Bagan test, where the *p*‐values were 0.54 for plasticity in germination probability; 0.00 for plasticity in germination phenology; 0.30 for plasticity in flowering phenology; 0.07 for plasticity in plant height; 0.34 for plasticity in flower abundance.
**Table S1:** Seed collection information. Latitude and longitude are in WGS84, and rounded to two digits. The exact location of *H. montanum* in Finland cannot be disclosed due to the species' conservation status (CR; Hyvärinen et al. 2019). The column “Origin” indicates whether the seeds were collected by us (Self coll. = self collected), or whether they were obtained from seed banks (Seed bank). The column “No. of mother inds.” indicates from how many plant individuals the seeds were sampled. The number of mother individuals from which seeds were sampled were unknown for populations originating from seedbanks, as they were bulk sampled.
**Table S2:** Summary statistics of response variables used in the models testing the effect of temperature on the traits. *N* = number of observations; SD = Standard deviation; Min = Minimum; Max = Maximum.
**Table S3:** Summary statistics of response and explanatory variables used in the models testing the effect of range position and environmental heterogeneity on trait plasticity. *N* = the number of observations; SD = standard deviation; Min = minimum; Max = Maximum; SHDI = Shannon diversity of land cover types; PAR = mean perimeter–area ratio of land cover patches; ARE = average roughness in topography; DRE = distance to range edge; DCE = distance to climatic edge.
**Table S4:** Model selection results for models testing the effect of range position and environmental heterogeneity on trait plasticity, with *species* included as a random effect to account nonindependence of observations originating from the same species. Each tested model included one variable from both groups, with one version of the model including their interaction. These models were compared to those that did not include *species* as a random effect. The models were fitted using *glmmTMB*() function in the “glmmTMB” R package (McGillycuddy et al. 2025). DRE = distance to distribution edge; DCE = distance to climatic edge; SHDI = Shannon diversity of land cover types; PAR = mean perimeter–area ratio of land cover patches; ARE = average roughness in topography; ΔAIC = difference in the AIC (Akaike's Information Criterion) value between the model compared to the best‐ranked model (ΔAIC = 0.0).
**Table S5:** Parameter estimates from the highest‐ranked models testing the effect of position within range and environmental heterogeneity on trait plasticity, with *species* included as a random effect. These results were compared with the results of models that did not include *species* as a random effect. The models were fitted using *glmmTMB*() function in the “glmmTMB” R package (McGillycuddy et al. 2025). SE = standard error, EH = environmental heterogeneity, RP = range position.
**Table S6:** Spearman correlation coefficients for the explanatory variables used in the models testing the effects of range position and environmental heterogeneity on trait plasticity. SHDI = Shannon diversity of land cover types; PAR = mean perimeter–area ratio of land cover patches; ARE = average roughness in topography; DRE = distance to range edge; DCE = distance to climatic edge.
**Table S7:** Summary statistics for the models testing the effect of range position and environmental heterogeneity on trait plasticity with less than 2 AIC (Akaike's Information Criterion) difference to the highest‐ranked model for each trait. SE = standard error.

## Data Availability

The data and code for reproducing the results are available for review purposes in dryad (https://doi.org/10.5061/dryad.r2280gbs0) and will be published upon acceptance of the manuscript.
