# Supplementary material for: Greater Thermal Plasticity Toward Heterogeneous Range‐Edge Environments of Three Hypericum Species
Source: Ecol Evol. 2026 Apr 30;16(5):e73486. doi: 10.1002/ece3.73486 (PMC13130348; doi:10.1002/ece3.73486)
Supplement: Supplementary file 1 — Figure S1: QQ‐plots of the models testing the effect of temperature on the traits. These were used to assess normality of residuals. A–C Germination probability; D–F germination phenology; and G–I flowering phenology for H. montanum (blue), H. perforatum (red), and H. maculatum (green), respectively J–K plant height and L–M flower abundance for H. montanum and H. perforatum . The normality of the residuals was additionally tested using Shapiro–Wilk test, where the p‐values for H. montanum , H. perforatum , and H. maculatum were 0.07, 0.11, and 0.31 for germination probability; 0.06, 0.00, and 0.00 for germination phenology; 0.03, 0.04, 0.00 for flowering phenology; 0.00, 0.45 for plant height; 0.00, 0.00 for flower abundance. Figure S2: Residuals versus fitted values for the models testing the effect of temperature on the traits. These were used to assess homoscedasticity of residuals. A–C Germination probability, D–F germination phenology; and G–I flowering phenology for H. montanum (blue), H. perforatum (red), and H. maculatum (green), respectively J,K plant height and L,M flower abundance for H. montanum and H. perforatum . The homoscedasticity of the residuals was additionally tested using Breusch‐Bagan test, where the p‐values for H. montanum , H. perforatum and H. maculatum were 0.00, 0.00, 0.00 for germination probability; 0.00, 0.00, 0.91 for germination phenology; 0.02, 0.00, 0.00 for flowering phenology; 0.00, 0.45 for plant height; 0.06, 0.00 for flower abundance. Figure S3: Loadings of bioclimatic variables on Principal Component (PC) 1 and 2. Loadings quantify the contribution of each variable to a principal component. Descriptions of bioclimatic variables can be found at https://www.worldclim.org/data/bioclim.html. Figure S4: QQ‐plots for the models testing the effect of position within range and environmental heterogeneity on trait plasticity. These were used to assess normality of residuals. The normality of the residuals was additionally tested usin [file ECE3-16-e73486-s001.docx]

**Supplementary information for:**

Koivusaari S. H. M., Hällfors, M. H., Hjort, J., Hyvärinen, M.-T., Levo, M., Luoto, M., Møller, C., Opedal, Ø., Pietikäinen, L., Romero-Bravo, A., Mattila, A. L. K. Greater thermal plasticity towards heterogeneous range-edge environments of three *Hypericum* species

**Contents:**

Figures S1-S5

Tables S1-S7

References


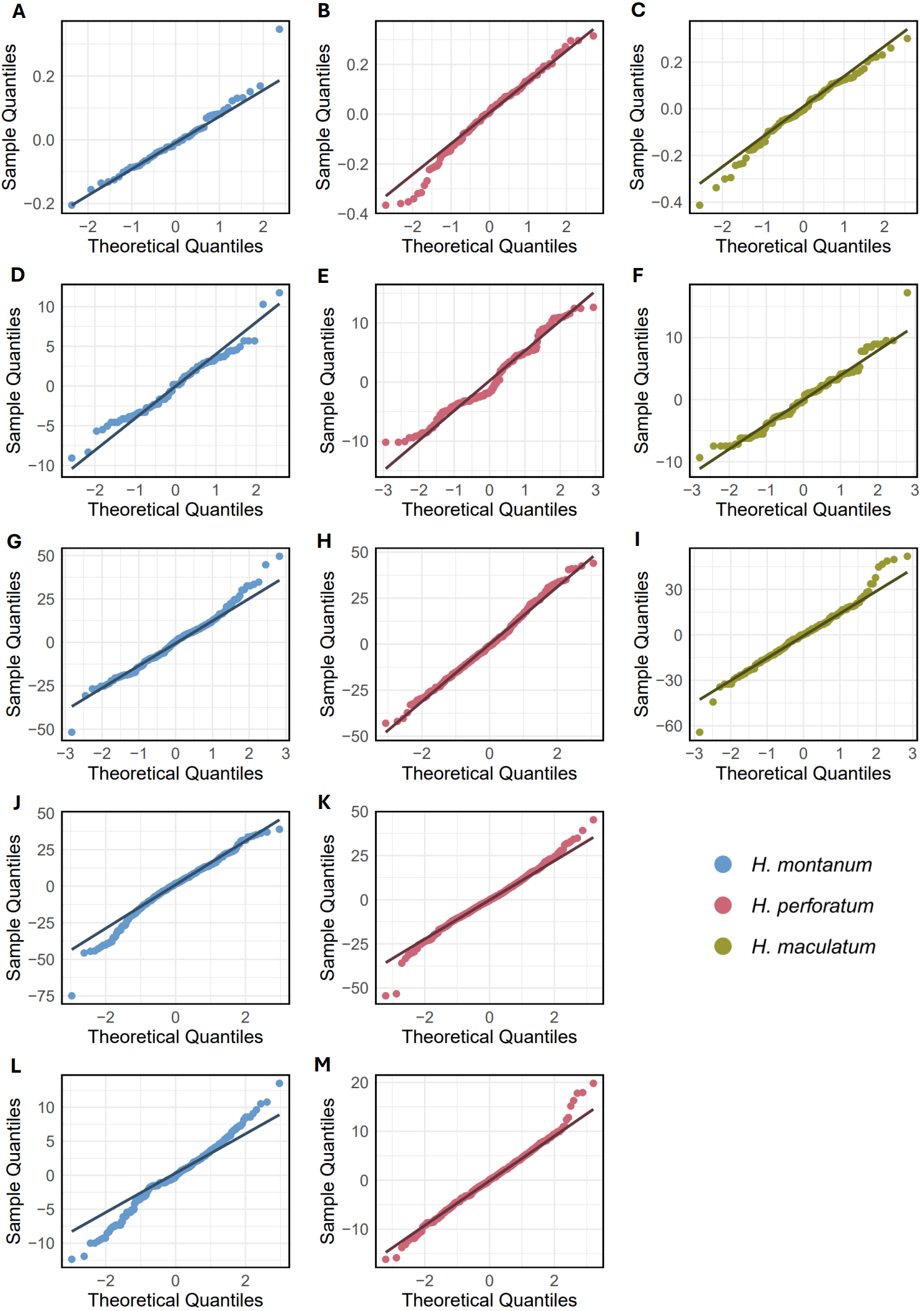


**Figure S1.** QQ-plots of the models testing the effect of temperature on the traits. These were used to assess normality of residuals. A-C Germination probability; D-F germination phenology; and G-I flowering phenology for *H. montanum* (blue), *H. perforatum* (red), and *H. maculatum* (green), respectively J-K plant height and L-M flower abundance for *H. montanum* and *H. perforatum*. The normality of the residuals was additionally tested using Shapiro-Wilk test, where the p-values for *H. montanum*, *H. perforatum*, and *H. maculatum* were 0.07, 0.11, and 0.31 for germination probability; 0.06, 0.00, and 0.00 for germination phenology; 0.03, 0.04, 0.00 for flowering phenology; 0.00, 0.45 for plant height; 0.00, 0.00 for flower abundance.


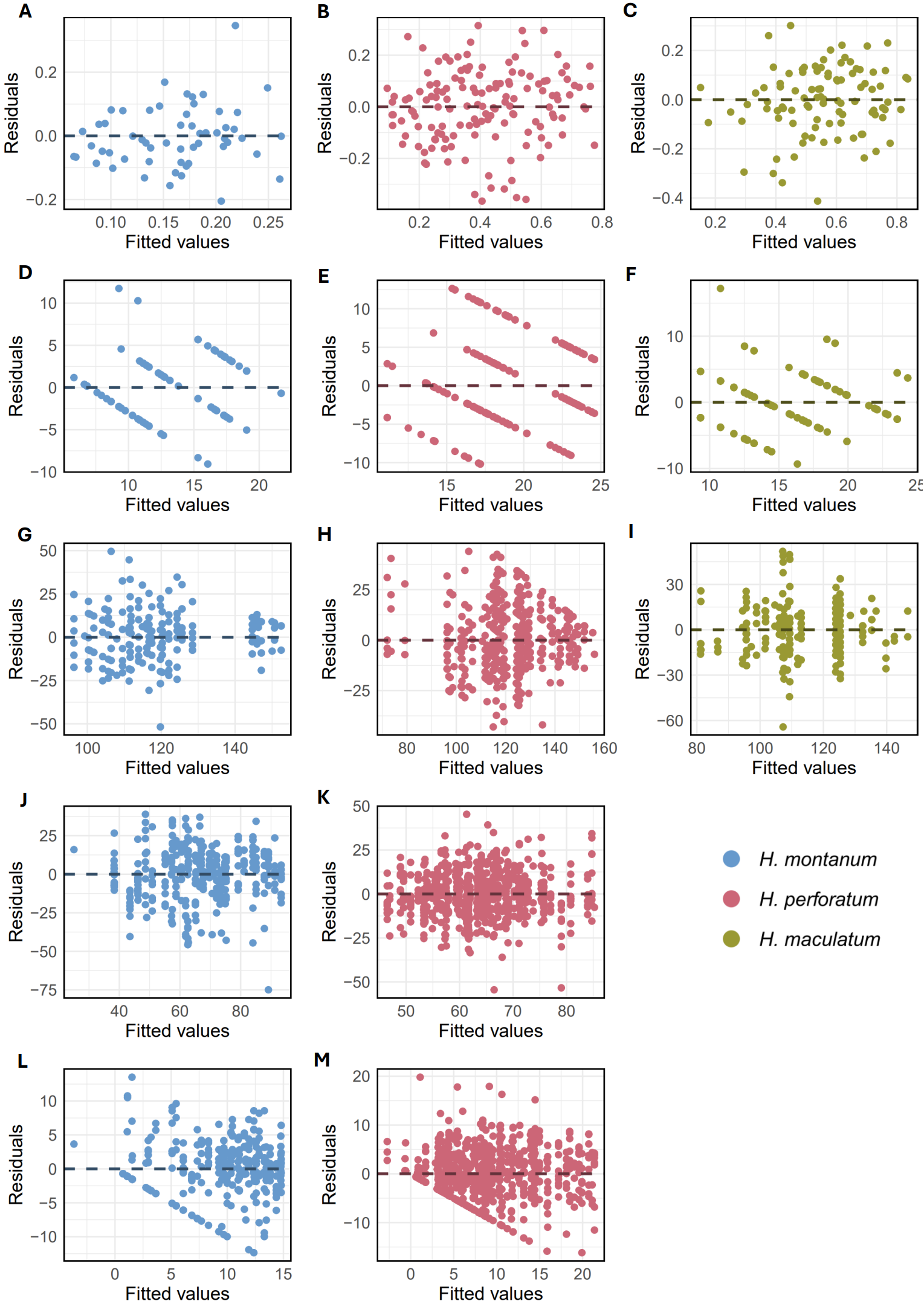


**Figure S2.** Residuals versus fitted values for the models testing the effect of temperature on the traits. These were used to assess homoscedasticity of residuals. A-C Germination probability, D-F germination phenology; and G-I flowering phenology for *H. montanum* (blue), *H. perforatum* (red), and *H. maculatum* (green), respectively J-K plant height and L-M flower abundance for *H. montanum* and *H. perforatum*. The homoscedasticity of the residuals was additionally tested using Breusch-Bagan test, where the p-values for *H. montanum*, *H. perforatum* and *H. maculatum* were 0.00, 0.00, 0.00 for germination probability; 0.00, 0.00, 0.91 for germination phenology; 0.02, 0.00, 0.00 for flowering phenology; 0.00, 0.45 for plant height; 0.06, 0.00 for flower abundance.

**
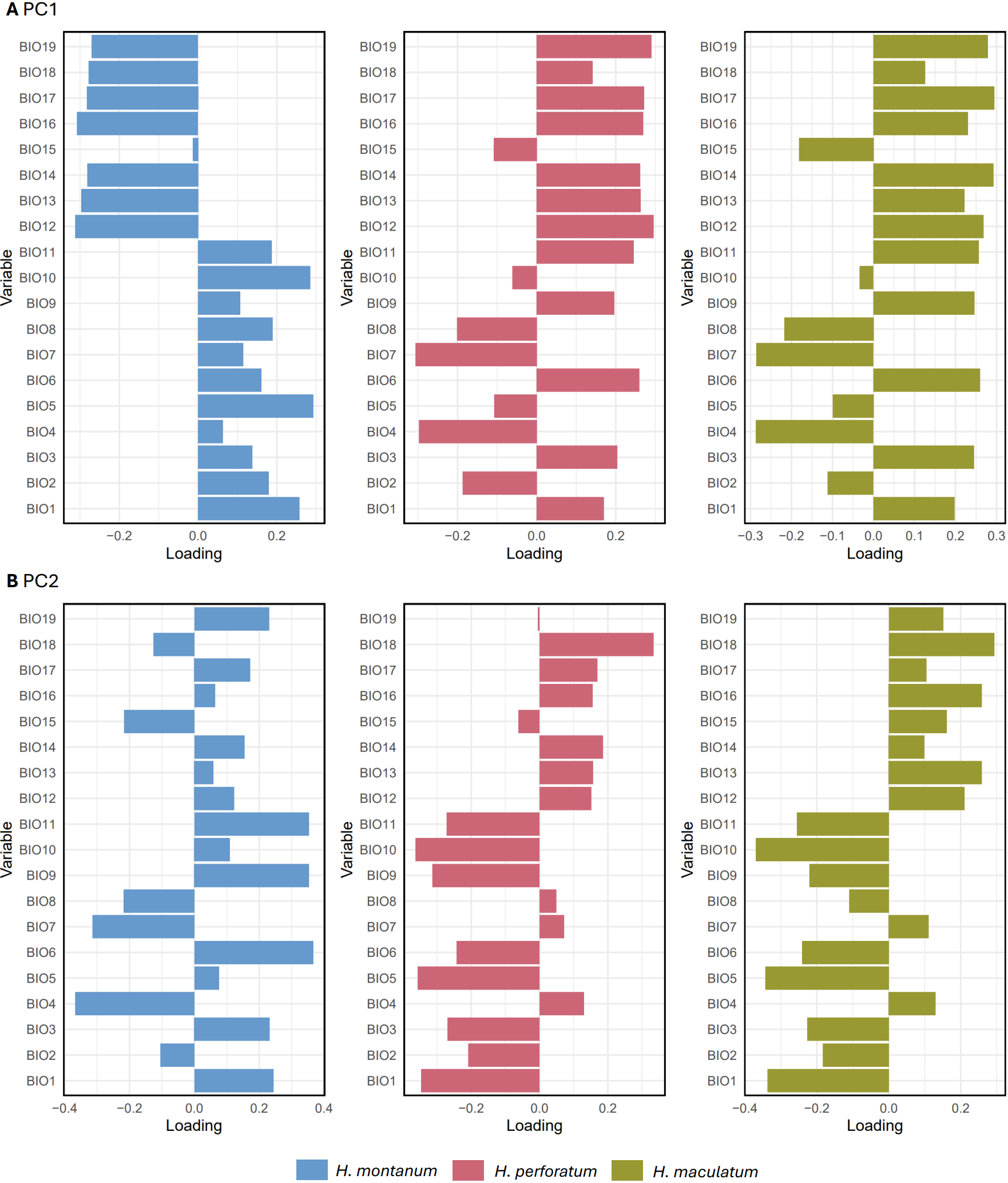
**

**Figure S3.** Loadings of bioclimatic variables on Principal Component (PC) 1 and 2. Loadings quantify the contribution of each variable to a principal component. Descriptions of bioclimatic variables can be found at https://www.worldclim.org/data/bioclim.html.

**
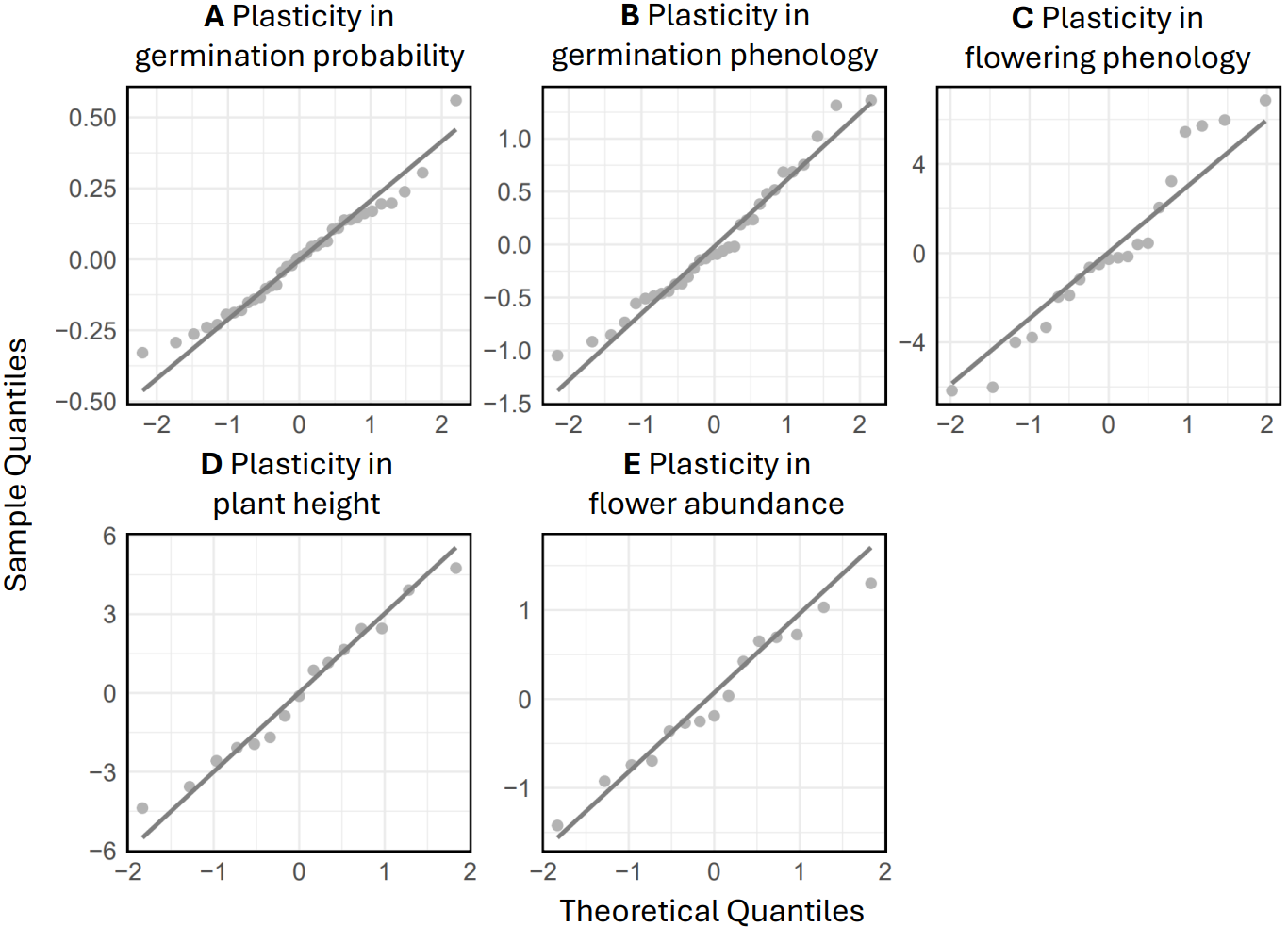
**

**Figure S4.** QQ-plots for the models testing the effect of position within range and environmental heterogeneity on trait plasticity. These were used to assess normality of residuals. The normality of the residuals was additionally tested using Shapiro-Wilk test, where the p-values were 0.42 for plasticity in germination probability; 0.38 for plasticity in germination phenology; 0.30 for plasticity in flowering phenology; 0.87 for plasticity in plant height; 0.87 for plasticity in flower abundance.

**
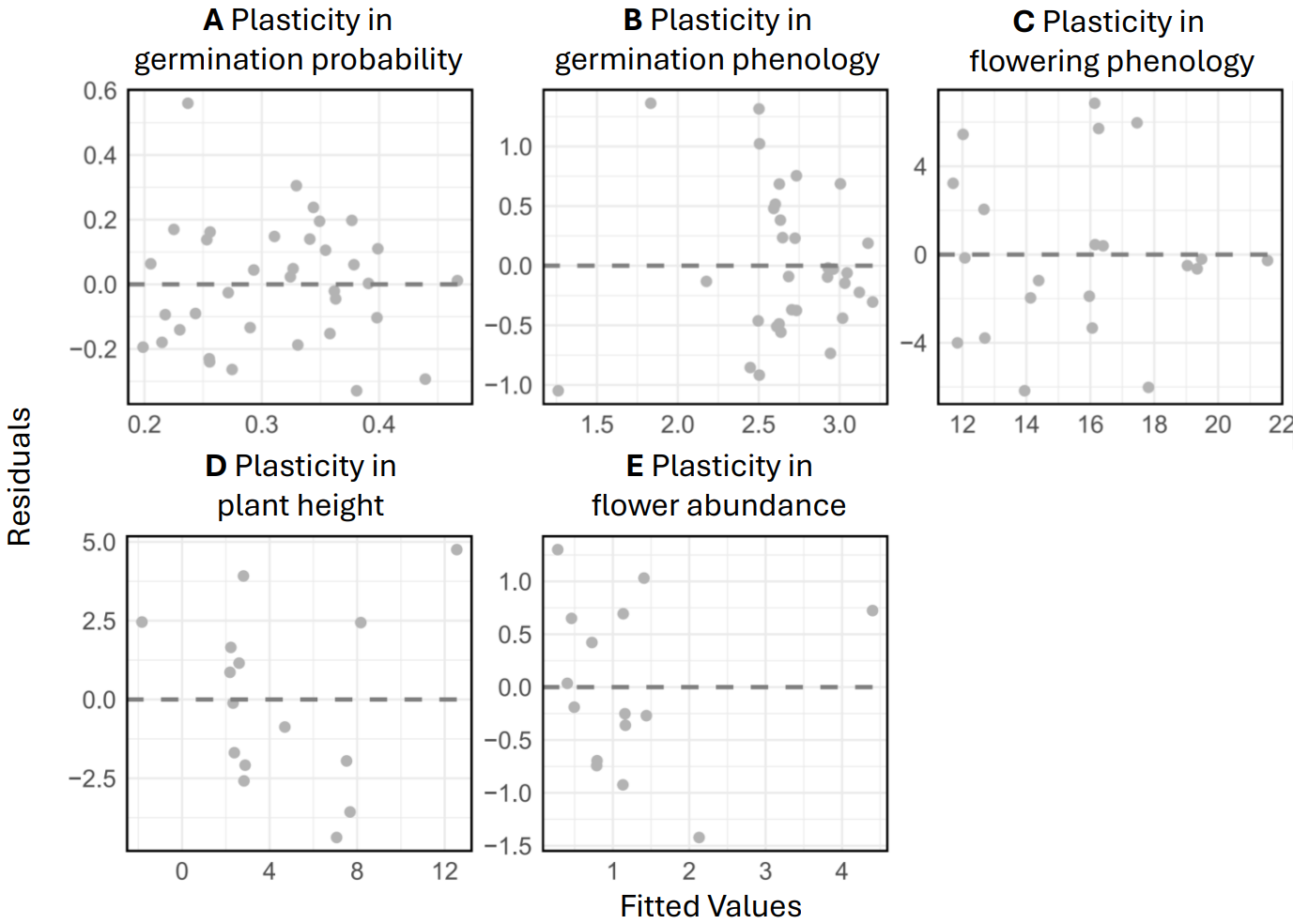
**

**Figure S5.** Residuals versus fitted values based on the models testing the effects of position within range and environmental heterogeneity on trait plasticity. These were used to assess homoscedasticity of residuals. The homoscedasticity of the residuals was additionally tested using Breusch-Bagan test, where the p-values were 0.54 for plasticity in germination probability; 0.00 for plasticity in germination phenology; 0.30 for plasticity in flowering phenology; 0.07 for plasticity in plant height; 0.34 for plasticity in flower abundance.

**Table S1.** Seed collection information. Latitude and longitude are in WGS84, and rounded to two digits. The exact location of *H. montanum* in Finland cannot be disclosed due to the species’ conservation status (CR; Hyvärinen et al. 2019). The column “Origin” indicates whether the seeds were collected by us (Self coll. = self collected), or whether they were obtained from seed banks (Seed bank). The column “No. of mother inds.” indicates from how many plant individuals the seeds were sampled. The number of mother individuals from which seeds were sampled were unknown for populations originating from seedbanks, as they were bulk sampled.

| **Species** | **Collection year** | **Country** | **Latitude** | **Longitude** | **Origin** | **No. of mother inds.** |
| --- | --- | --- | --- | --- | --- | --- |
| *H. montanum* | 2020 | Sweden | 57.79 | 18.89 | Self coll. | 29 |
|  | 2017 | Poland | 52.70 | 23.85 | Seed bank | NA |
|  | 2021 | France | 44.42 | 5.46 | Self coll. | 8 |
|  | 2019 | France | 44.18 | 3.43 | Seed bank | NA |
|  | 2021 | Finland | 60 | 24 | Self coll. | NA |
|  | 2018 | Austria | 47.81 | 13.12 | Seed bank | NA |
|  | 2018 | Switzerland | 47.01 | 7.00 | Seed bank | NA |
|  | 1998 | UK | 51.46 | -2.63 | Seed bank | NA |
| *H. perforatum* | 2021 | Finland | 60.21 | 24.96 | Self coll. | 52 |
|  | 2021 | Finland | 59.83 | 22.93 | Self coll. | 50 |
|  | 2021 | France | 48.40 | 2.63 | Self coll. | 15 |
|  | 2021 | France | 48.83 | 2.11 | Self coll. | 30 |
|  | 2021 | UK | 50.9 | -0.04 | Self coll. | NA |
|  | 2021 | UK | 50.88 | -0.02 | Self coll. | NA |
|  | 2021 | UK | 50.87 | 0.00 | Self coll. | NA |
|  | 2021 | Belgium | 50.08 | 4.55 | Self coll. | 40 |
|  | 2021 | France | 44.76 | 6.28 | Self coll. | 38 |
|  | 2021 | France | 44.45 | 5.44 | Self coll. | 23 |
|  | 2020 | Slovenia | 45.64 | 14.23 | Seed bank | NA |
|  | 2019 | Norway | 59.95 | 10.84 | Seed bank | NA |
|  | 2020 | Germany | 52.56 | 13.59 | Seed bank | NA |
|  | 2020 | Italy | 45.70 | 6.95 | Seed bank | NA |
|  | 2021 | Belgium | 50.89 | 4.30 | Self coll. | 40 |
|  | 2007 | UK | 50.90 | -0.05 | Seed bank | NA |
|  | 2011 | UK | 50.88 | -0.02 | Seed bank | NA |
|  | 2021 | Belgium | 49.51 | 5.48 | Self coll. | 40 |
| *H. maculatum* | 2020 | Finland | 60.26 | 23.60 | Self coll. | 57 |
|  | 2020 | Belgium | 50.50 | 6.25 | Self coll. | 30 |
|  | 2018 | Austria | 48.11 | 13.29 | Seed bank | NA |
|  | 2021 | France | 46.14 | 6.59 | Self coll. | 21 |
|  | 2008 | Italy | 46.12 | 9.57 | Seed bank | NA |
|  | 2020 | Finland | 60.18 | 24.70 | Self coll. | 56 |
|  | 2020 | Sweden | 55.70 | 13.45 | Self coll | 10 |
|  | 2019 | Norway | 63.54 | 10.65 | Seed bank | NA |
|  | 2019 | Estonia | 59.48 | 25.01 | Seed bank | NA |
|  | 2018 | Austria | 47.34 | 11.12 | Seed bank | NA |
|  | 2019 | Switzerland | 46.93 | 6.57 | Seed bank | NA |
|  | 2019 | France | 45.90 | 6.43 | Seed bank | NA |

**Table S2.** Summary statistics of response variables used in the models testing the effect of temperature on the traits. N = number of observations; SD = Standard deviation; Min = Minimum; Max = Maximum.

| **Trait** | **Species** | **N** | **Mean** | **SD** | **Min** | **Median** | **Max** |
| --- | --- | --- | --- | --- | --- | --- | --- |
| Germination proportion | H. montanum | 56 | 0.16 | 0.11 | 0.00 | 0.15 | 0.57 |
|  | H. perforatum | 144 | 0.41 | 0.23 | 0.00 | 0.42 | 0.92 |
|  | H. maculatum | 96 | 0.55 | 0.21 | 0.00 | 0.56 | 1.00 |
| Germination phenology | H. montanum | 197 | 23.84 | 11.72 | 7 | 21 | 35 |
|  | H. perforatum | 1397 | 31.63 | 7.05 | 7 | 35 | 35 |
|  | H. maculatum | 1239 | 32.00 | 7.39 | 7 | 35 | 35 |
| Flowering phenology | H. montanum | 347 | 118.04 | 20.77 | 68 | 117 | 159 |
|  | H. perforatum | 744 | 120.88 | 22.74 | 65 | 121 | 159 |
|  | H. maculatum | 263 | 113.13 | 22.05 | 43 | 114 | 159 |
| Plant height | H. montanum | 347 | 66.54 | 22.55 | 3.10 | 70.5 | 119.40 |
|  | H. perforatum | 744 | 64.08 | 14.82 | 12.00 | 64.3 | 119.00 |
| Flower abundance | H. montanum | 347 | 110.57 | 101.14 | 0 | 104 | 478 |
|  | H. perforatum | 744 | 129.71 | 166.61 | 0 | 67 | 878 |

**Table S3.** Summary statistics of response and explanatory variables used in the models testing the effect of range position and environmental heterogeneity on trait plasticity. N = the number of observations; SD = standard deviation; Min = minimum; Max = Maximum; SHDI = Shannon diversity of land cover types; PAR = mean perimeter-area ratio of land cover patches; ARE = average roughness in topography; DRE = distance to range edge; DCE = distance to climatic edge.

| **Variable** | **N** | **Mean** | **SD** | **Min** | **Median** | **Max** |
| --- | --- | --- | --- | --- | --- | --- |
| Plasticity in germination probability | 37 | 0.31 | 0.20 | 0.00 | 0.34 | 0.80 |
| Plasticity in germination phenology | 33 | 2.68 | 0.72 | 0.21 | 2.89 | 3.82 |
| Plasticity in flowering phenology | 22 | 15.56 | 4.63 | 7.77 | 15.02 | 23.43 |
| Plasticity in plant height | 16 | 4.83 | 4.64 | 0.25 | 3.79 | 17.31 |
| Plasticity in flower abundance | 16 | 1.20 | 1.23 | 0.05 | 1.01 | 5.12 |
| SHDI | 38 | 1.37 | 0.34 | 0.57 | 1.38 | 2.04 |
| PAR | 38 | 0.31 | 0.02 | 0.27 | 0.31 | 0.34 |
| ARE | 37 | 30.21 | 32.06 | 3.05 | 14.16 | 123.33 |
| DRE | 38 | 1177364.61 | 539863.83 | 260585.66 | 1062595.77 | 2030743.07 |
| DCE | 38 | 4.1 | 0.96 | 1.94 | 3.96 | 5.91 |

**Table S4.** Model selection results for models testing the effect of range position and environmental heterogeneity on trait plasticity, with *species* included as a random effect to account nonindependence of observations originating from the same species. Each tested model included one variable from both groups, with one version of the model including their interaction. These models were compared to those that did not include *species* as a random effect. The models were fitted using *glmmTMB()* function in the ‘glmmTMB’ R package (McGillycuddy et al., 2025). DRE = distance to distribution edge; DCE = distance to climatic edge; SHDI = Shannon diversity of land cover types; PAR = mean perimeter-area ratio of land cover patches; ARE = average roughness in topography; ΔAIC = difference in the AIC (Akaike’s Information Criterion) value between the model compared to the best-ranked model (ΔAIC = 0.0).

| **Range position metric used** | **Landscape heterogeneity metric used** | **Interaction included** | **Plasticity in germination probability** | **Plasticity in germination phenology** | **Plasticity in flowering phenology** | **Plasticity in plant height** | **Plasticity in flower abundance** |
| --- | --- | --- | --- | --- | --- | --- | --- |
|  |  |  | **ΔAIC** | **ΔAIC** | **ΔAIC** | **ΔAIC** | **ΔAIC** |
| DRE | SHDI | No | 1.6 | 5.0 | 6.4 | 10.2 | 10.5 |
| DRE | SHDI | Yes | 1.8 | 3.8 | 6.9 | 12.0 | 11.7 |
| DRE | PAR | No | 2.5 | 5.0 | 3.2 | 9.1 | 11.4 |
| DRE | PAR | Yes | 1.9 | 4.4 | 3.1 | 0.0 | 7.8 |
| DRE | ARE | No | 2.5 | 5.1 | 0.8 | 10.3 | 11.7 |
| DRE | ARE | Yes | 4.2 | 5.4 | 2.7 | 0.3 | 0.0 |
| DCE | SHDI | No | 0.0 | 5.7 | 6.0 | 12.0 | 11.2 |
| DCE | SHDI | Yes | 2.0 | 3.9 | 7.7 | 11.3 | 13.2 |
| DCE | PAR | No | 0.7 | 5.6 | 3.6 | 10.3 | 11.6 |
| DCE | PAR | Yes | 2.1 | 7.4 | 5.4 | 12.2 | 13.2 |
| DCE | ARE | No | 0.5 | 5.7 | 0.0 | 11.9 | 12.1 |
| DCE | ARE | Yes | 3.4 | 0.0 | 1.3 | 12.5 | 12.7 |

**Table S5.** Parameter estimates from the highest-ranked models testing the effect of position within range and environmental heterogeneity on trait plasticity, with *species* included as a random effect. These results were compared with the results of models that did not include *species* as a random effect. The models were fitted using *glmmTMB()* function in the ‘glmmTMB’ R package (McGillycuddy et al., 2025). SE = standard error, EH = environmental heterogeneity, RP = range position.

| **Trait** | **Term** | **Estimate** | **SE** | **z-value** | **p-value** |
| --- | --- | --- | --- | --- | --- |
| Plasticity in germination probability | Intercept | 0.310 | 0.037 | 8.384 | <0.001 |
|  | EH | -0.040 | 0.032 | -1.272 | 0.203 |
|  | RP | -0.054 | 0.033 | -1.636 | 0.102 |
| Plasticity in germination phenology | Intercept | 2.660 | 0.108 | 24.722 | <0.001 |
|  | EH | -0.147 | 0.112 | -1.317 | 0.188 |
|  | RP | 0.078 | 0.112 | 0.697 | 0.486 |
|  | EH:RP | -0.296 | 0.100 | -2.959 | <0.01 |
| Plasticity in flowering phenology | Intercept | 15.578 | 0.818 | 19.049 | <0.001 |
|  | EH | 2.524 | 0.839 | 3.009 | <0.01 |
|  | RP | -1.071 | 0.839 | -1.277 | 0.202 |
| Plasticity in plant height | Intercept | 4.075 | 0.688 | 5.920 | <0.001 |
|  | EH | 1.660 | 0.722 | 2.298 | <0.05 |
|  | RP | -2.146 | 0.729 | -2.943 | <0.01 |
|  | EH:RP | -2.679 | 0.661 | -4.053 | <0.001 |
| Plasticity in flower abundance | Intercept | 1.216 | 0.195 | 6.227 | <0.001 |
|  | EH | 0.439 | 0.205 | 2.134 | <0.05 |
|  | RP | -0.385 | 0.206 | -1.872 | <0.1 |
|  | EH:RP | -1.212 | 0.257 | -4.725 | <0.001 |

**Table S6.** Spearman correlation coefficients for the explanatory variables used in the models testing the effects of range position and environmental heterogeneity on trait plasticity. SHDI = Shannon diversity of land cover types; PAR = mean perimeter-area ratio of land cover patches; ARE = average roughness in topography; DRE = distance to range edge; DCE = distance to climatic edge.

|  | **SHDI** | **PAR** | **ARE** | **DRE** | **DCE** |
| --- | --- | --- | --- | --- | --- |
| SHDI | 1 | 0.16 | -0.08 | 0.01 | -0.17 |
| PAR |  | 1 | 0.05 | -0.14 | -0.01 |
| ARE |  |  | 1 | -0.35 | -0.06 |
| DRE |  |  |  | 1 | -0.40 |
| DCE |  |  |  |  | 1 |

**Table S7.** Summary statistics for the models testing the effect of range position and environmental heterogeneity on trait plasticity with less than 2 AIC (Akaike’s Information Criterion) difference to the highest-ranked model for each trait. SE = standard error.

| **Trait** | **Term** | **Estimate** | **SE** | **t-value** | **p-value** |
| --- | --- | --- | --- | --- | --- |
| Plasticity in germination probability | Intercept | 0.312 | 0.033 | 9.389 | <0.001 |
|  | SHDI | -0.035 | 0.034 | -1.025 | 0.313 |
|  | DRE | 0.044 | 0.034 | 1.308 | 0.200 |
| Plasticity in germination probability | Intercept | 0.309 | 0.033 | 9.338 | <0.001 |
|  | SHDI | -0.038 | 0.034 | -1.133 | 0.266 |
|  | DRE | 0.040 | 0.034 | 1.201 | 0.239 |
|  | SHDI:DRE | -0.041 | 0.033 | -1.269 | 0.214 |
| Plasticity in germination probability | Intercept | 0.308 | 0.033 | 9.314 | <0.001 |
|  | PAR | -0.010 | 0.034 | -0.290 | 0.774 |
|  | DRE | 0.036 | 0.034 | 1.053 | 0.300 |
|  | PAR:DRE | -0.041 | 0.027 | -1.528 | 0.136 |
| Plasticity in germination probability | Intercept | 0.312 | 0.033 | 9.515 | <0.001 |
|  | PAR | -0.031 | 0.034 | -0.929 | 0.360 |
|  | DCE | -0.064 | 0.034 | -1.890 | <0.1 |
| Plasticity in germination probability | Intercept | 0.317 | 0.034 | 9.410 | <0.001 |
|  | PAR | -0.030 | 0.034 | -0.891 | 0.380 |
|  | DCE | -0.063 | 0.034 | -1.860 | <0.1 |
|  | PAR:DCE | 0.031 | 0.043 | 0.716 | 0.479 |
| Plasticity in germination probability | Intercept | 0.312 | 0.033 | 9.409 | <0.001 |
|  | ARE | 0.011 | 0.034 | 0.333 | 0.742 |
|  | DCE | -0.058 | 0.034 | -1.702 | <0.1 |
| Plasticity in flowering phenology | Intercept | 15.578 | 0.900 | 17.305 | <0.001 |
|  | ARE | 2.560 | 0.922 | 2.775 | <0.05 |
|  | DRE | 0.759 | 0.922 | 0.823 | 0.421 |
| Plasticity in flowering phenology | Intercept | 15.607 | 0.895 | 17.446 | <0.001 |
|  | ARE | 2.270 | 0.975 | 2.327 | <0.05 |
|  | DCE | -1.213 | 0.935 | -1.297 | 0.212 |
|  | ARE:DCE | 0.739 | 0.970 | 0.762 | 0.456 |
| Plasticity in plant height | Intercept | 4.075 | 0.804 | 5.070 | <0.001 |
|  | PAR | 1.660 | 0.844 | 1.968 | <0.1 |
|  | DRE | -2.146 | 0.852 | -2.520 | <0.05 |
|  | PAR:DRE | -2.679 | 0.772 | -3.471 | <0.01 |

**References**

Hyvärinen, E., Juslén, A. K., Kemppainen, E., Uddström, A., & Liukko, U.-M. (Toimittajat) (2019). *Suomen lajien uhanalaisuus 2019 - Punainen kirja: The 2019 Red List of Finnish Species*. Ympäristöministeriö & Suomen ympäristökeskus. <http://hdl.handle.net/10138/299501>

McGillycuddy, M., Popovic, G., Bolker, B. M., & Warton, D. I. (2025). Parsimoniously Fitting Large Multivariate Random Effects in glmmTMB. *Journal of Statistical Software*, *112*, 1–19. https://doi.org/10.18637/jss.v112.i01
